# Using MR-chordless circuits for efficient enumeration of autocatalytic cores in large chemical reaction networks

**DOI:** 10.1186/s13321-026-01240-3

**Published:** 2026-08-20

**Authors:** Richard Golnik, Nicola Vassena, Peter F. Stadler, Thomas Gatter

**Affiliations:** 1 Bioinformatics Group, Department of Computer Science, Leipzig University, Härtelstraße 16–18, 04107 Leipzig, Germany; 2 Interdisciplinary Center for Bioinformatics, Leipzig University, 04107 Leipzig, Germany; 3 Center for Scalabale Data Analytics and Artificial Intelligence, Leipzig University, 04107 Leipzig, Germany; 4 Zuse School for Embedded and Composite Artificial Intelligence (SECAI), Leipzig University, 04107 Leipzig, Germany; 5 Max Planck Institute for Mathematics in the Sciences, Inselstraße 22, 04103 Leipzig, Germany; 6 Department of Theoretical Chemistry, University of Vienna, Währingerstraße 17, 1090 Wien, Austria; 7 Facultad de Ciencias, Universidad National de Colombia, Bogotá, Colombia; 8 Center for non-coding RNA in Technology and Health, University of Copenhagen, Ridebanevej 9, 1870 Frederiksberg, Denmark; 9 Santa Fe Institute, 1399 Hyde Park Rd., Santa Fe, NM 87501 USA

**Keywords:** Autocatalysis, Graph-theoretic algorithm, Exhaustive enumeration, Metabolic networks, Irreversible reactions

## Abstract

Autocatalysis is an important property of chemical reaction networks (CRNs) that is particularly prevalent in metabolic networks. A set of well-defined *autocatalytic cores* prominently features minimal subsystems that determine the autocatalytic capabilities. Recently, a graph-theoretic characterization has become available that enabled the enumeration of moderate-sized autocatalytic cores in real-life metabolic networks. Such an approach relies on enumerating and properly assembling elementary circuits in the bipartite graph associated to a CRN. Here, we improve on this approach in two ways: (1) We elaborate on algorithms for the enumeration of elementary circuits restricted to so-called MR-chordless circuits. These circuits do not have a chord from a Metabolite to a Reaction vertex, and are the only candidates to find autocatalytic cores. (2) We interleave our new algorithm with tests for autocatalysis to further limit the number of circuits that need to be stored for the construction of autocatalytic cores more complex than elementary MR-chordless circuits. Combined, these innovations achieve a performance gain of several orders of magnitude and make it possible to exhaustively enumerate all autocatalytic cores in real-life metabolic reaction networks comprising several hundred metabolites and reactions. Importantly, we find that reaction networks with irreversible reactions contain complex autocatalytic cores comprising more than a single “cycle with an ear”. Such structures exceed the established classification of autocatalytic cores for fully reversible networks into five types.

**Scientific contribution**

We developed a new graph-theoretic algorithm for enumerating autocatalytic cores that can handle large genome-scale metabolic models. Implemented in the Python program autogatito, it is up to four orders of magnitude faster than previous methods. Applications to large metabolic network models that involve both reversible and nonreversible reactions reveal that more complex autocatalytic cores exist than predicted by existing classification schemes for reversible reactions.

**Supplementary Information:**

The online version contains supplementary material available at 10.1186/s13321-026-01240-3.

## Introduction

**Fig. 1:**
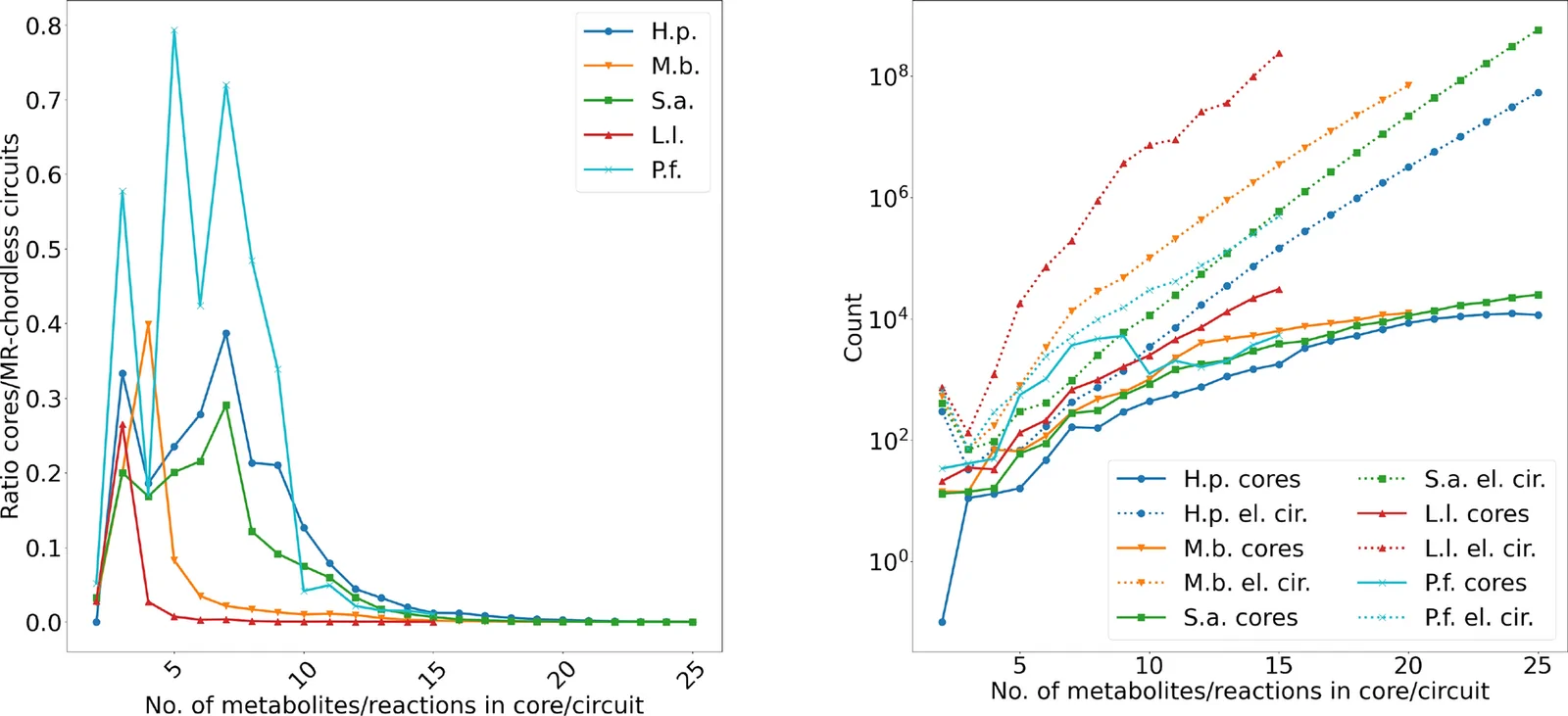
Ratio (**left**) of the number of autocatalytic cores over the number of elementary circuits and the absolute counts (**right**) of both, depicted as a function of the number of metabolites in the autocatalytic cores and elementary circuits, indicated on the horizontal axis. The reference metabolic networks are *Helicobacter pylori* [18] (H.p., 208 metabolites and 333 reactions), *Methanosarcina barkeri* (M.b., 249 metabolites and 402 reactions [19]), *Staphylococcus aureus* (S.a., 270 metabolites and 456 reactions [20]), *Lactococcus lactis* (L.l., 264 metabolites and 448 reactions [21]), and *Plasmodium falciparum Ljungdahlii* (P.f., 297 metabolites and 530 reactions [22]), after removal of the set of most highly interconnecting metabolites. For details on the networks, see the supplementary information

Autocatalytic reaction networks comprise sets of reactions in which the products collectively catalyze their own formation. Apart from food and waste metabolites, they form self-maintaining sub-structures in chemical reaction networks (CRNs) that are of key interest for the analysis of contemporary metabolic networks [1–4] and for studies of the transition between non-living and living matter [5–8]. Until recently, several alternative definitions of structural autocatalysis in chemical reaction networks were in use [2, 9–11], complicating the comparison of computational results. The definition given in [10], which exhibits several convenient mathematical properties [12], has been widely accepted in recent years. It also forms the basis of practical algorithms to analyze empirical networks, including a heuristic based on combinations of cycles [13], an ILP enumerating minimal autocatalytic subnetworks [11], a SAT-solver based approach [14], and a combinatorial algorithm to enumerate irreducible autocatalytic subnetworks [15]. The latter approach, implemented in the Python project autogato [15] is based on the explicit enumeration of elementary circuits in the directed bipartite König representation of the CRN. This representation assigns one class of vertices to species (Metabolites) and the other to Reactions. There is a directed edge (MR) from a species *x* to a reaction *r* if *x* is a reactant of *r*, and a directed edge (RM) from a reaction *r* to a species *x* if *x* is a product of *r*. While the number of elementary circuits in a digraph can grow super-exponentially with size, e.g., in complete digraphs, the number of autocatalytic cores may stabilize depending on the size and connectedness of the network (see Fig. 1). The reason – informally – is that the minimality condition inherent to autocatalytic cores becomes more difficult to satisfy for larger subsystems.

The autogato software includes a modified algorithm to determine only autocatalytic cores instead of enumerating all irreducible autocatalytic subsystems [15]. It enumerates all elementary circuits up to a particular, user-defined size, engaging a modification of Johnson’s algorithm [16]. The computational cost of enumerating a very large number of elementary circuits to find a relatively small amount of cores, however, becomes disproportionate (Fig. 1). It was already noted in [15] that elementary circuits contributing to autocatalytic cores cannot have directed chords connecting a reactant (metabolite) vertex to a reaction vertex, so called MR-chords. We therefore call such circuits MR-chordless. This observation suggests replacing the enumeration of all elementary circuits with a dedicated enumeration procedure for MR-chordless circuits. We therefore developed a specific algorithm to enumerate this type of elementary circuits in bipartite graphs. To this end, we use ideas similar to the approach of Dias et al. [17] for enumerating chordless circuits in general.

This contribution is organized as follows: we briefly summarize the key mathematical results underlying the combinatorial algorithm for computing autocatalytic cores with autogato [15] in section Theoretical background. In section The algorithm we describe our algorithm and in particular the necessary modifications to the approach of Dias et al. [17] for enumerating general chordless circuits towards enumerating only MR-chordless elementary circuits in bipartite digraphs. We also provide a self-contained proof that our algorithm, indeed, identifies only MR-chordless elementary circuits, determines them exhaustively in a given bipartite graph, and avoids duplications. Finally, we introduce a modification of our enumeration algorithm designed for the efficient detection of autocatalytic cores. This variant, termed autogatito, is provided as part of the autogato Python project. Extensive benchmarking in section Benchmarking shows that our algorithm enumerating MR-chordless elementary circuits directly outperforms the combination of Johnson’s algorithm with post-processing by up to 4 orders of magnitude. The integration of this feature into autogato [15], together with avoiding the enumeration of larger MR-chordless elementary circuits that cannot be part of cores because they contain the vertex set of a smaller autocatalytic elementary circuit, results in a further performance improvement.

## Theoretical background

A *chemical reaction network* (CRN) Γ is a pair of sets Γ=(X,R), where *X* is the set of *species* or *metabolites*, and *R* is the set of *reactions*. Any reaction r∈R is an ordered association between nonnegative combinations of the species:

1 $$\begin{aligned} r:\quad \sum _{x\in X} s^-_{xr} x \quad \rightarrow \quad \sum _{x\in X} s^+_{xr}x, \end{aligned}$$

where the nonnegative constants $s^{-}_{xr}$ and $s^{+}_{xr}$ are the *stoichiometric coefficients*. A species *x* is a *reactant* of the reaction *r* if $s^-_{xr}>0$ and a *product* of *r* if $s^+_{xr}>0$. For simplicity of presentation, we exclude *explicit catalysts* in this contribution. That is, we assume that there is no species *x* such that $s^-_{xr}>0$ and $s^+_{xr}>0$ for some reaction *r*, no species is both reactant and product of the same reaction. In this case, the associated *stoichiometric matrix* $\textbf{S}\in \mathbb {Z}^{|X|\times |R|}$, defined as $s_{xr}:=s^{+}_{xr}-s^{-}_{xr}$, fully encodes the network Γ. In particular, $s_{xr}=s^{+}_{xr}$, whenever *x* is a product of *r*, $s_{xr}=-s^{-}_{xr}$ whenever *x* is a reactant of *r*. Otherwise $s_{xr}=0$. It is possible to extend the theory of autocatalytic subsystems to CRN with explicit catalysis, see [23] for details.

We write $X^{\prime }$ and $R^{\prime }$ to refer to subsets of *X* and *R*. The set of metabolites participating in a set of reactions $R^{\prime }$ is denoted by $$X(R'):=\{x \in X \ | \ \exists \ r\in R: s_{xr}\ne 0 \}$$. The network Γ is conveniently represented by its bipartite *König graph* $$\textbf{K}=(V,E)$$ with vertex set V:=X∪R and edge set $$E:=E_1\cup E_2$$, where $$(x,r)\in E_1$$ iff $$s_{xr}<0$$ and $$(r,x)\in E_2$$ iff $$s_{xr}>0$$. We call $$E_1$$ the *metabolite-to-reactions* (MR) edges, and $$E_2$$ the *reaction-to-metabolite* (RM) edges. Similarly, $$E'_j$$ refers to subsets of $$E_j$$, j=1,2. In the following, we use some notation and terminology that is standard in graph theory. We refer to the SI (Sect. *Graph Theory*) for definitions of these concepts.

**Fig. 2:**
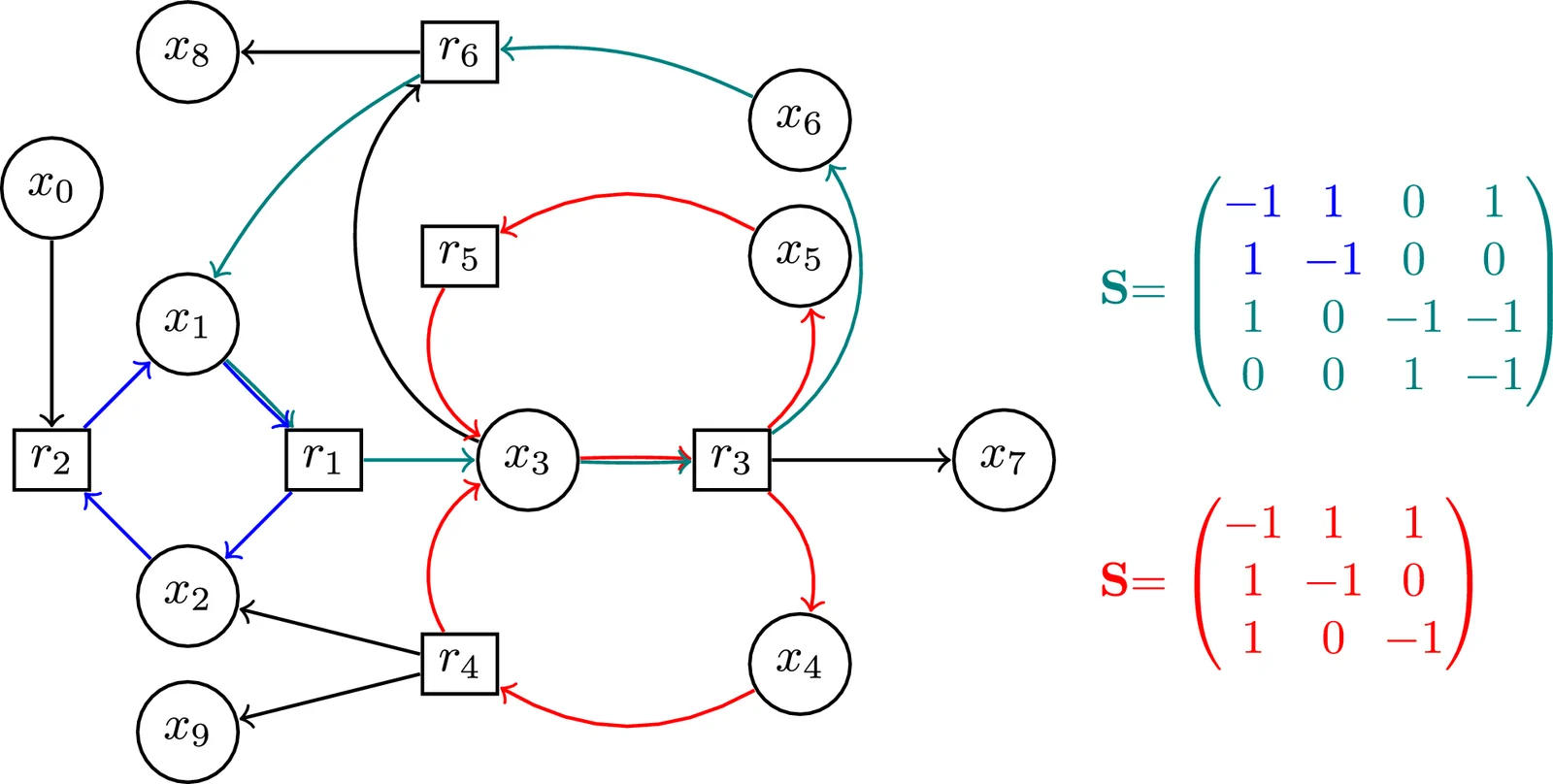
**Left:** Graphic illustration of a chemical reaction network in its bipartite König representation. Stoichiometric coefficients only take values in $\{0,1\}$. An elementary circuit with an MR-chord from $x_3$ to $r_6$ is depicted in teal, while an MR-chordless elementary circuit in blue. The blue and the teal elementary circuits, together, yield a fluffle. However, this is not an induced fluffle, precisely due to the MR-chord from $x_3$ to $r_6$. An induced fluffle, which is, in addition, an autocatalytic core, is marked in red. **Upper right:** The stoichiometric matrix associated with the fluffle composed of the teal and blue elementary circuits. With a proper column ordering, the MR-chord in the elementary circuit appears as a negative off-diagonal entry. **Lower right:** The stoichiometric matrix associated with the induced fluffle (and autocatalytic core) is marked in red. With the proper column ordering, the matrix is a Metzler matrix, i.e., with nonnegative off-diagonal entries

We use the notation $$\textbf{S}'$$ to refer to the submatrix of $$\textbf{S}$$ indexed by species-rows $X^{\prime }\subseteq X$ and reaction-columns $R^{\prime }\subseteq R$. A vector *v* is called *strictly positive*, denoted by $${\textbf {v}}>0$$ if all of its entries are strictly positive. A submatrix $$\textbf{S}'$$ is *autocatalytic* [10] if

(A1): for all columns $r\in R^{\prime }$ there are rows $x,y\in X^{\prime }$ such that $$s_{xr}<0$$ and $$s_{yr}>0$$, and
(A2): there is a strictly positive vector $\textbf{v}>0$ such that $\textbf{S}'\textbf{v}>0$ is again strictly positive.

An *autocatalytic core* (equivalently a PAC in [14]) is an autocatalytic matrix $$\textbf{S}'$$ that possesses no proper autocatalytic submatrix. As shown in [10, 12], there is a 1-1 correspondence of species and reactions in $$\textbf{S}'$$ such that each $x\in X^{\prime }$ is the unique reactant in $X^{\prime }$ of a uniquely defined reaction $$r_x\in R'$$. Therefore, $$\textbf{S}'$$ is square and the columns of $$\textbf{S}'$$ can be reordered such that the resulting matrix $$\textbf{A}$$ is a matrix with strictly negative diagonal entries and nonnegative off-diagonal entries. Up to the proper ordering of the columns, the latter property defines $$\textbf{S}'$$ as a *Metzler matrix*. Further, such Metzler matrix is irreducible. The closely related *minimal autocatalytic subnetwork* (MAS) [11] is a subnetwork $\left(X\left(R^{\prime }\right),R^{\prime }\right)$ that contains an autocatalytic core and $R^{\prime }$ is inclusion-minimal with this property. Hence, the reaction set $R^{\prime }$ of a MAS is the reaction set of at least one autocatalytic core. The enumeration of autocatalytic cores thus also yields a complete list of MAS.

The square sub-matrices of the stoichiometric matrix which, up to re-ordering of their columns, can be written as Metzler matrices, are in 1-1 correspondence with the induced *fluffles* of the bipartite König graph $\textbf{K}$ [15]. A *Fluffle* is a subgraph $G=(X'\cup R',E'_1\cup E'_2)$ of $\textbf{K}$ satisfying the following properties:

(i): *G* is a strongly connected block,
(ii): $|R^{\prime }\left|=\right|X^{\prime }|$, and
(iii): every $x\in X^{\prime }$ has out-degree 1 and every $r\in R^{\prime }$ has in-degree 1.

See the *Graph Theory* section in the SI for a self-contained presentation of the standard concepts of *induced subgraphs*, *strong-connectedness*, and *in- and out-degree*. The crucial observation here is that the subgraph $\textbf{K}[X(C)\cup R(C)]$ induced by an elementary circuit *C* is a fluffle if and only if *C* is MR-chordless, i.e., if there is no edge $(x,r)\in E_1$ which is not in *E*(*C*). A graphical summary about the relationship of (induced) fluffles and Metzler matrices is shown in Fig. 2. Finally, with an iterative argument based on the *ear-decomposition* of strong blocks, it can be shown that all induced fluffles are a superposition of MR-chordless circuits. The detailed arguments are published in [15]. The search for candidate autocatalytic cores, therefore, can be restricted to elementary circuits without MR-chords and their superpositions. Moreover, by minimality of cores, autocatalytic elementary circuits do not need to be combined with other elementary circuits.

## The Algorithm

A more efficient enumeration of autocatalytic cores should thus be achievable by replacing Johnson’s algorithm for listing all elementary circuits [24] by a variant that produces MR-chordless circuits only. A natural starting point for this endeavor is an algorithm by Dias et al. for the enumeration of chord-free circuits [17]. In the following section, we describe the modifications necessary to adapt this algorithm for our purposes.

### Preprocessing

The algorithm described in [17] forbids all kinds of chords. In bipartite digraphs, however, there are two classes of directed edges, distinguished by vertices that form the heads and tails, respectively. The implementation below is independent of the identifiers of the two disjoint vertex sets. Nevertheless, in the setting of CRNs we refer to the two types of edges, and thus also of chords, as metabolite-to-reaction (MR) and reaction-to-metabolite (RM). For better readability, we refer to elements of the two disjoint vertex sets *X* and *R* as metabolites and reactions, respectively.

In fact, the assumption of a bipartite graph considerably simplifies some special cases that have to be accommodated in the general case:

1. Self-loops cannot occur in a bipartite graph and thus do not have to be handled.
2. Elementary circuits of length 2, so-called *digons*, are chordless and thus can be easily collected in a preprocessing step (see Alg. 1). However, Digons cannot be removed from the original graph, in contrast to [17], because its MR-edge, might appear as an edge along an elementary circuit, while the corresponding RM-edge constitutes an acceptable chord.
3. Bipartite graphs are triangle-free and hence quadrangles are the smallest elementary circuits that can exhibit an MR-chord. Quadrangles, therefore, are the smallest structures detected by the main enumeration function of our algorithm.

The graph $\textbf{K}$ is first decomposed into its strongly connected components (SCCs), which can be analyzed separately. Since SCCs with less than 4 vertices cannot encounter two reactions and reactants, they are omitted. For each larger component, our goal is to enumerate all MR-chordless circuits. The key building blocks are *stems*, defined as directed paths of length three that initiate and terminate in metabolite vertices, *x* and *y*, and have an interior reaction vertex, denoted as *r*. For a given reaction *r*, the set of stems, denoted as $\mathcal {S}_r$, is obtained as the cartesian product of the predecessor and successor vertices of *r*, i.e., the reactants and products of the reaction *r* in a CRN. An element of $\mathcal {S}_r$ is denoted as S=(x,r,y). Alg. 1 generates all stems for a reaction *r* in the strongly connected component *C* and then calls MRChordlessCircuitSearch$(\texttt {C}, \texttt {S}, \mathcal {D})$ for each $S\in \mathcal {S}_r$, (Alg. 2) to enumerate all MR-chordless circuits that contain *S*, where $\mathcal {D}$ is an optional set containing reactions to be ignored.

**Algorithm 1:**
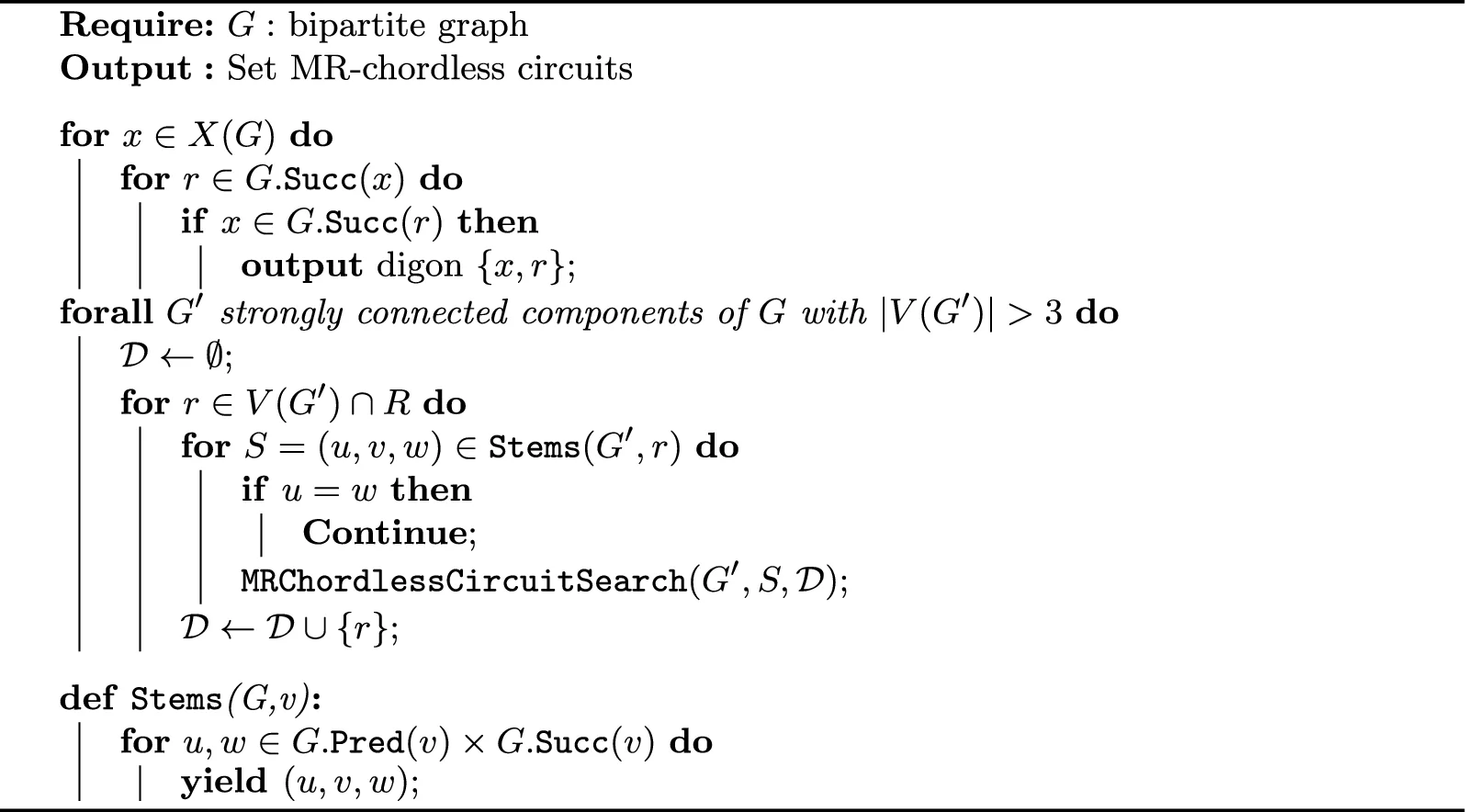
Top-level strategy for enumerating MR-chordless circuits. The enumeration of circuits starting from a stem *S* is handled in procedure MRChordlessCircuitSearch(), described in Alg. 2 MR Chordless Circuit Search, which also produces the output.

To avoid generating a circuit *C* more than once, we observe that every elementary circuit has a first reaction vertex *r* with respect to any arbitrary ordering < of the vertices in *R*. If *r* appears in the stem S=(x,r,y), and all subsequent reaction vertices $r^{\prime }$ satisfy $r^{\prime }>r$, then *C* is generated exactly once. This can be achieved by restricting the successor function to $r^{\prime }>r$ while the stem S=(x,r,y) is processed. For practical purposes, this order does not need to be computed explicitly. It suffices to either modify *G* by removing *r* before executing the next steps, or, as shown in the pseudocode, to establish a list of already visited reactions $$\mathcal {D}$$ that are no longer considered by the successor function.

### MR-chordless circuit search

Alg. 2 represents the core function of the MR-chordless circuit search. Dias et al. [17] enumerated paths via depth-first-search and took advantage of a map to identify vertices that were allowed to be added to the path $\textbf{P}$. In particular, a node could only be part of a chordless circuit if it was a neighbor of exactly one vertex on the present path, i.e., its predecessor in the directed case.

In our setting, however, two of these data structures are required, one for metabolites (*B*, “*backwards*”) and one for reactions (*F*, “*forwards*”), both initialized with value 0 for each key. Values are updated initially for the reaction *r* and the metabolites *x* and *y*; subsequently, *F* and *B* are updated exclusively when a vertex is appended to or removed from the path. The direction of the update depends on the type of vertex appended or removed. A reaction updates all its predecessors, i.e., all its reactant species, while a metabolite updates all its successors, i.e., all reactions it is a reactant for. Upon attachment, *F* and *B* are modified by +1 and upon removal by -1. If F[r]=1 after initialization, then both *x* and *y* are reactants for *r* and the search for this stem is terminated since all elementary circuits containing this stem also exhibit the MR-chord (*y*, *r*).

**Algorithm 2:**
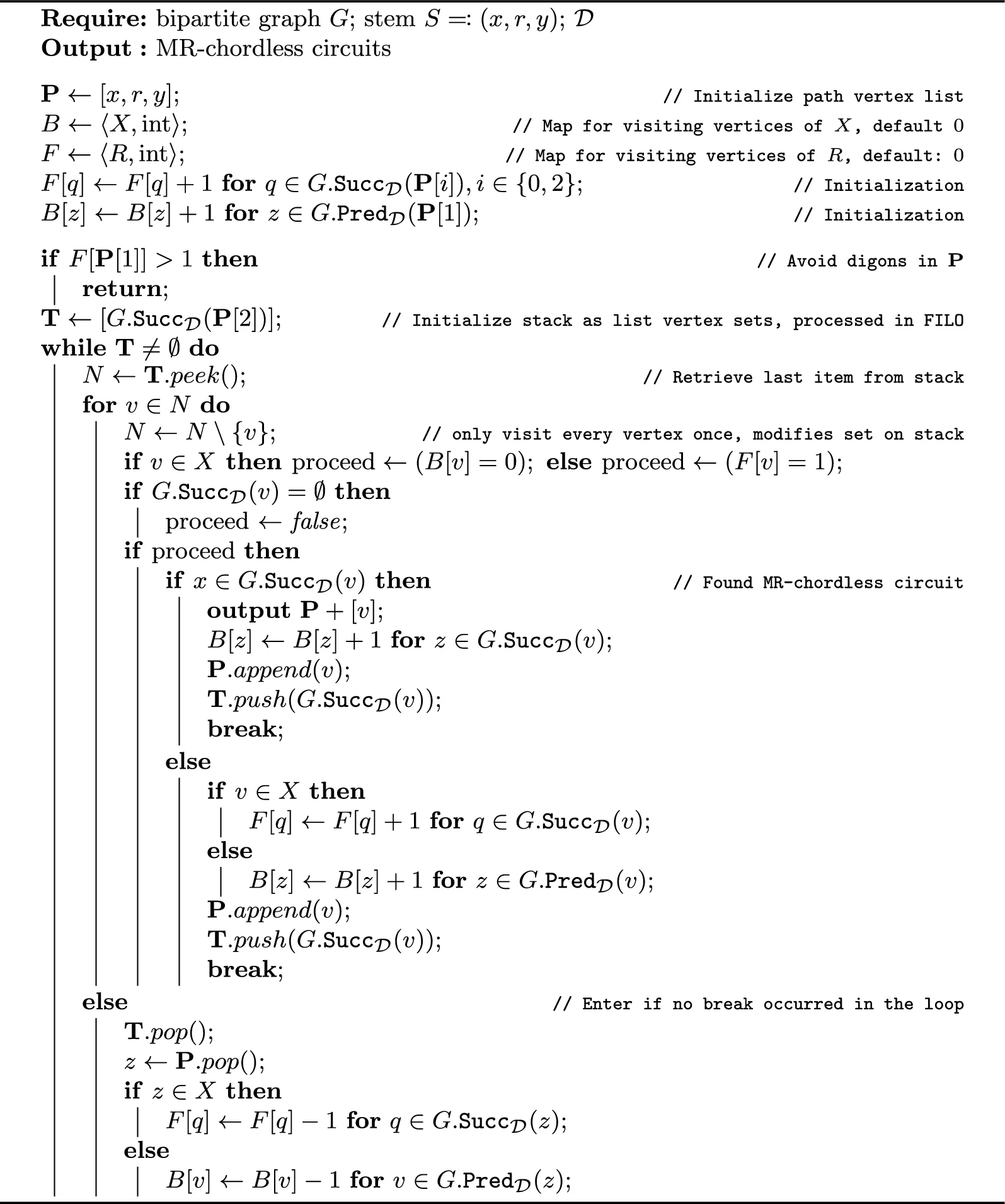
MRChordlessCircuitSearch$(\texttt {G}, \texttt {S}, \mathcal {D})$ enumerates all MR-chordless circuits with stem *S* in a strongly connected bipartite digraph excluding reaction vertices in $\mathcal{D}$. We define function $G.\texttt{Succ}_\mathcal{D}(x)$ such that it returns all successors $r \notin \mathcal{D}$ of x∈X in *G*. Although not essential for the correctness of the algorithms, we also define the restricted predecessor function $G.\texttt{Pred}_\mathcal{D}(x)$ that returns all predecessors $r \notin \mathcal{D}$ of x∈X in *G*

A stack $\textbf{T}$, composed of sets of vertices that remain to be visited along the nascent path $\textbf{P}$ is utilized for efficient vertex traversal, modifying the concept of classic *iterative* depth-first-search as follows. Vertices can be visited more than once if the path to reach them is changed. Hence, we cannot rely on global visited markers. Instead, sets of successors that have yet to be visited for each node in the current path are tracked in the stack. $\textbf{T}$ itself is processed in first-in-last-out (FILO) order. Any visited successor of the current terminating vertex of the growing path $\textbf{P}$ is removed from the leading set of $\textbf{T}$.

Upon initialization, the set of all successors of *y* is pushed onto $\textbf{T}$. All successors in the leading set of $\textbf{T}$ are visited consecutively until a valid amendment is found, which is added to the nascent path while the set of its successors is loaded onto the stack in turn. Upon backtracking, i.e., if the leading set of $\textbf{T}$ is exhausted, it is removed from $\textbf{T}$, and the algorithm continues by processing the new top element of $\textbf{T}$.

A reaction $r_i$ is processed only if $F[r_i]=1$, implying that exactly one of the metabolites on the current path $\textbf{P}$ is a reactant for $r_i$, while a metabolite $x_i$ is processed only if $B[x_i]=0$, in particular if it is not a reactant for any reaction that has already been added to $\textbf{P}$. The initial vertex *x* is therefore never visited due to initialization of *B* (see Lemma 2, Claim 2 and Claim 4). In addition, *r* can also never be revisited since F[r]=1 by initialization and any additional traversal would require another predecessor of *r* and hence F[r]≥2, which violates general processing requirement for reactions. For *y* we obtain B[y]=1 directly after addition of the necessary fourth vertex to obtain at least a quadrangle, which prevents a second visit.

Further processing of vertices depends on their particular type. For a reaction vertex $r_i$, it is initially verified if *x* is a successor (product) for $r_i$ since this would imply that an MR-chordless elementary circuit has been found. The corresponding circuit is subsequently returned to the main function. Since RM-chords are specifically allowed, we extend the search by adding $r_i$ to the current path, and the set of vertices consisting of successors of $r_i$ is pushed to the stack, as it is performed in case that *x* is not a successor of $r_i$. Whenever none of the out-neighbors of the ultimate vertex *v* of $\textbf{P}$ can extend the current path, *v* is removed from $\textbf{P}$, its set of successors removed from the stack, and *F* and *B* are updated as described previously.

### Correctness of the algorithms

Let *G* be a bipartite digraph G:=(X∪R,E) composed of two disjoint vertex sets *X* and *R*. We denote a subgraph $G^{\prime }:=\left(X^{\prime }\cup R^{\prime },E^{\prime }\right)$ of *G* with $$X'\subseteq X, R'\subseteq R$$, and $E^{\prime }\subseteq E$ by $G^{\prime }\subseteq G$. By a slight abuse of notation we write $S\subseteq G^{\prime }$ for a stem S:=(x,r,y) if $$G[\{x,r,y\}]\subseteq G'$$. We assume throughout that $G^{\prime }\subseteq G$ be a strongly connected subgraph of *G*. We start with some simple observations

#### Lemma 1

(i) Alg. 1 enumerates each digon exactly once.
(ii) Let $G^{\prime }\subseteq G$ be a strongly connected subgraph of *G* with $r\in R(G^{\prime })$ and $S\subseteq G^{\prime }$. Then Stems($G^{\prime },r$) enumerates all stems of the form S=(x,r,y).

#### Proof

(i) All digons are detected in the pre-processing step of Alg. 1. Since it iterates only over the x∈X(G), i.e., the species vertices, successors of *x* can only be reaction vertices r∈R(G). If the backwards edge (*r*, *x*) exists, a digon is detected and returned. Thus, all digons are detected. Assume now, there is a digon $\left(x^{\prime },r^{\prime }\right)$ that is detected twice. This, however, implies that either the outer for-loop running over all species vertices enumerates $x^{\prime }$ twice or, for $x^{\prime }$, the inner for-loop enumerates $r^{\prime }$ twice, which is not possible because of the unique enumeration scheme of a for-loop.

(ii) By construction, the function Stems($G^{\prime },r$) returns all stems containing a given $r\in R(G^{\prime })$. □

In the following notation, we drop the delete set $\mathcal {D}$ as a mere implementation detail for brevity.

The function MRChordlessCircuitSearch($S,G^{\prime }$) enumerates lists $$\textbf{Q}$$ of vertices that form paths with the additional property that there is an edge from the final to the starting vertex. During construction, the vertex lists $$\textbf{Q}$$ correspond to paths without this final edge. We therefore write $$(\textbf{Q},x)$$ for the extension of the list $$\textbf{Q}$$ by a vertex in the following.

#### Lemma 2

Let $G^{\prime }$ be a strongly connected graph, $S:=\left(x,r,y\right)\subset G^{\prime }$, x≠y and let $$\textbf{Q}$$ be a list returned by MRChordlessCircuitSearch($S,G^{\prime }$). Then $$(\textbf{Q},x)$$ is an MR chord-free elementary circuit.

#### Proof

By definition, S:=(x,r,y) with x=y corresponds to a digon. Since the digons are detected in the preprocessing step, a call of MRChordlessCircuitSearch($S,G^{\prime }$) with x=y would be redundant. In the following $$\textbf{Q}$$ denotes a sequence of vertices returned by MRChordlessCircuitSearch($S,G^{\prime }$). We first show that $$(\textbf{Q},x)$$ is a closed walk and $$\textbf{Q}$$ is a path:

**Claim 1.** MRChordlessCircuitSearch($S,G^{\prime }$) never removes *x* or *r* from $$\textbf{P}$$ if $S:=\left(x,r,y\right)\subset G^{\prime }$ and x≠y.

Upon initialization, the stack $\textbf{T}$ contains the set ${{\,\textrm{succ}\,}}(y)$ of all successors of *y*. Since $\textbf{T}$ is processed in FILO order, the bottom elements of $\textbf{T}$ will always be ${{\,\textrm{succ}\,}}(y)$. Thus, if *y* is removed from $\textbf{P}$, then ${{\,\textrm{succ}\,}}(y)$ is removed from $\textbf{T}$, we obtain $\textbf{T}=\emptyset$ and hence Alg. 2 terminates. Thus, neither *x* nor *r* are ever removed from $\textbf{P}$. ⋄

**Claim 2.** *If* $S\subset G^{\prime }$*, then the sequence of vertices* $$\textbf{Q} = (x_1,r_1,x_2,r_2, \ldots , x_k,r_k)$$ *forms a walk that satisfies* $$x_1=x, r_1=r$$, and $$x_2=y$$.

A direct consequence of Claim 1 is that each sequence enumerated by Alg. 2 contains the path *S*. Moreover, since $$r_k\in R$$ by construction, *S* is a proper subsequence of each $$\textbf{Q}$$. The sequence of vertices $$\textbf{Q}$$ forms a path in $G^{\prime }$ by construction: Denoting by $$\textbf{P}=(v_1, v_2, v_3,,\ldots , v_l)$$ the nascent sequence in MRChordlessCircuitSearch(), we see immediately that it is a walk since only vertices are added that are adjacent to the terminal vertex of $$\textbf{P}$$. ⋄

We write $S\subset \textbf{Q}$ as a shorthand for Claim 2.

**Claim 3.*** The extension* $(\textbf{Q},x)$ *of* $\textbf{Q}$ *is a closed walk*.

Let S:=(x,r,y) be a stem with $S\subset G^{\prime }$. By Claim 2, $$\textbf{P}=(x,r,y,r_2,\dots ,x_k)$$ is walk and the extension $$(\textbf{P}, r_k)$$ is returned if only if $$(r_k, x)\in E(G')$$. In addition, visiting $$r_k$$ implies $$r_k\in {{\,\textrm{succ}\,}}(x_k), v_l=x_k$$ and that there is an edge $$(x_k, r_k)\in E(G')$$. Thus, $$(\textbf{Q},x)=(\textbf{P}, r_k, x)$$ is a closed walk. ⋄

**Claim 4.** *No vertex of S is visited twice along the walk* $\textbf{Q}$.

Recall that *S* is a path since x≠y. After initialization, F[r]=1 and since *x* is never removed from $$\textbf{P}$$ by Claim 1, *r* is never added again to $$\textbf{P}$$. Conversely, B[x]=1 and since *r* is never removed from $$\textbf{P}$$ (Claim 1), *x* cannot be visited again on $$\textbf{P}$$. Finally, since $G^{\prime }$ is strongly connected, $${{\,\textrm{succ}\,}}(y)\ne \emptyset$$ and since *G* is bipartite, $G^{\prime }$ is also bipartite and hence (*x*, *y*) is not an edge. If $\left(x,r^{\prime }\right)\in E\left(G^{\prime }\right)$ for all $$r'\in {{\,\textrm{succ}\,}}(y)$$ then *y* is removed from $$\textbf{P}$$, $${{\,\textrm{succ}\,}}(y)$$ is removed from $$\textbf{T}$$, and MRChordlessCircuitSearch($S,G^{\prime }$) terminates since $$\textbf{T}=\emptyset$$. In any other case, at least one $$r'\in R(G'): r'\ne r$$ is added to $$\textbf{P}$$, hence B[y]=1. Then *y* can only be visited again if $r^{\prime }$ is removed from $$\textbf{P}$$, which would imply $$\textbf{P}=S$$. We can then repeat this argument for all $$r''\in {{\,\textrm{succ}\,}}(y)$$ with $\left(x,r^{\prime \prime }\right)\notin E\left(G^{\prime }\right)$. ⋄

**Claim 5.** $\textbf{Q}$ *is a path.*

Assume that there is a $q\in R(G^{\prime })$, q∉S that was added twice to $$\textbf{Q}=(\textbf{P},r_k)$$. Then there are edges $$(x_i,q)\in E(\textbf{Q})$$ and $$(x_j,q)\in E(\textbf{Q})$$ such that F[q]=1 as $$x_i$$ was added to $$\textbf{P}$$ and F[q]=2 as $$x_j$$ was added to $$\textbf{P}$$. The latter is not possible, however. Assume now there is a $z\in X(G^{\prime })$ with z∉S that was added twice to $$\textbf{P}$$. Then there is an edge $$(z,r_i)\in E(\textbf{P})$$ and hence B[z]=1 upon adding of $$r_i$$ to $$\textbf{P}$$. This, however, impedes adding *z* again until $$r_i$$ is removed from $$\textbf{P}$$, yielding B[z]=0 again. If $$r_i$$ is removed, the same argument holds for any other $$r_j \in R(G')$$ with $$r_i\ne r_j$$, and $$(z, r_j)\in E(G')$$, and thus no such *z* can exist. ⋄

Since $\textbf{Q}=(\textbf{P},r_k)$ is a path (Claim 5) starting with *x* and $(\textbf{Q},x)$ is a closed walk (Claim 3) $(\textbf{Q},x)$ is in fact an elementary circuit.

**Claim 6.** *The elementary circuit *$(\textbf{Q},x)$ *has no MR-chord.*

Assume there is an edge $(z,q)\in G[(\textbf{Q}, x)]$ with $(z, q)\notin E(\textbf{Q},x)$. If z=x then F[q]=1 upon initialization. Since q≠r, *q* cannot be visited until *x* is removed from $\textbf{P}$ because B[x]=1 upon initialization. However, by Claim 1, *x* is never removed from $\textbf{P}$, hence *q* can never be visited, and, thus, $q\notin \textbf{Q}$, a contradiction. Hence z≠x. Then there is an edge $(w,q)\in E((\textbf{Q}, x))$ with z≠w. Assume first that *z* was added to $\textbf{P}$ before *q*. Then *z* was also added before *w* because *q* is the direct successor of *w* on $\textbf{P}$. Hence, F[q]≥1 as *z* is added to $\textbf{P}$ and F[q]≥2 as *w* is added to $\textbf{P}$, which forbids visiting and appending *q* to $\textbf{P}$, hence yields a contradiction. Therefore, *q* was added before *z* and hence B[z]≥1 as *q* is added to $\textbf{P}$, violating the condition B[z]=0 for processing and appending *z*. Thus $z\notin \textbf{Q}$, a contradiction. It follows that $(\textbf{Q},x)$ is MR-chordless. □

#### Lemma 3

MRChordlessCircuitSearch($S,G^{\prime }$) enumerates all MR-chordless circuits containing $S\subset G^{\prime }$. With the restricted successor function it enumerates all MR-chordless circuits containing $S\subset G^{\prime }$ with reaction vertices $$r_j\ge r$$.

#### Proof

Let *C* be an MR-chordless circuit. We can write $$C=(\textbf{Q},x)$$ where $$\textbf{Q}$$ is a path of the form $$\textbf{Q}=(x_1, r_1, x_2, \ldots , r_k)$$ with $$x_1=x$$, $$r_1=r$$, and $$x_2=y$$. Moreover, $$x \in X(\textbf{Q})$$ has out-degree 1 and each $$r\in R(\textbf{Q})$$ has in-degree 1. Upon initialization of MRChordlessCircuitSearch($S,G^{\prime }$), we have $$r_2\in {{\,\textrm{succ}\,}}(y)$$ and $$F[r_2]=1$$ and hence the function will eventually visit $$r_2$$. If $$r_2=r_k$$ we have found $$\textbf{Q} = (\textbf{P},r_2)$$. Otherwise, $$r_2$$ is added to $$\textbf{P}$$ and $$(r_2,x_3)\in E(\textbf{Q})\subseteq E(G')$$ implies $$x_3\in {{\,\textrm{succ}\,}}(r_2)$$. Since $$x_3$$ has out-degree 1 by virtue of *C* being an MR-chordless circuit, we have $$B[x_3]=0$$ and thus the function will eventually visit $$x_3$$ and add to $$\textbf{P}$$. Repeating this argument until $$r_k$$ is reached shows that the function MRChordlessCircuitSearch() with input $\left(S,G^{\prime }\right)$ will eventually construct and return $$\textbf{Q}=(\textbf{P}, r_k)$$. The argument remains the same if $$r_j\ge r_1$$ and $${{\,\textrm{succ}\,}}(x,r_1)$$ returns the list of all successors *r* of x∈X satisfying $$r\ge r_1$$. □

#### Lemma 4

For given $S\subset G^{\prime }$, each path $$\textbf{Q}$$ returned by MRChordlessCircuitSearch($S,G^{\prime }$) is unique.

#### Proof

We proceed by induction. Let $S:=(x_1, r_1, x_2)$. For the base case k=2 we consider $\textbf{P}'_{r^i_2} :=(S, r^i_2)$ for $r^i_2, r^j_2\in {{\,\textrm{succ}\,}}(x_2)$. We have $\textbf{P}'_{r^i_2} \ne \textbf{P}'_{r^j_2}$ if and only if $r^i_2 \ne r^j_2$, and thus also $\textbf{Q}_{r^i_2} \ne \textbf{Q}_{r^j_2}$ for $(r_2^i,x_1),(r_2^j,x_1)\in E(G')$ with $r^i_2 \ne r^j_2$. Induction hypothesis: all paths $\textbf{P}= (x_1, r_1, x_2, \ldots , x_k)$ with $k\le \ell$ are produced only once. Then it suffices to show that each extension to $(\textbf{P},r_k,x_{k+1})$ is constructed only once. In the first step $\textbf{P}$ can be extended by any $x^i_{k+1}\in {{\,\textrm{succ}\,}}(r_k)$ with $B[x^i_{k+1}]=0$. We have $\textbf{P}_{x^i_{k+1}} \ne \textbf{P}_{x^j_{k+1}}$ for $x^i_{k+1}\ne x^j_{k+1}$ and $(r_k, x^i_{k+1}), (r_k, x^j_{k+1})\in E(G')$ and $B[x^i_{k+1}] = B[x^j_{k+1}] = 0$. Similarly, let $\textbf{P}=(x_1, r_1, \ldots r_k, x_{k+1})$, then each $\textbf{P}_{r^i_{k+1}}\ne \textbf{P}_{r^j_{k+1}}$ for $r^i_{k+1}, r^j_{k+1} \in {{\,\textrm{succ}\,}}(x_{k+1})$ with $r^i_{k+1}\ne r^j_{k+1}$ and $F[r^i_{k+1}] = F[r^j_{k+1}] = 1$. This implies $\textbf{Q}_{r^i_{k+1}} \ne \textbf{Q}_{r^j_{k+1}}$. □

We are now in the position to prove the main result of this section.

#### Theorem 5

Let *G* be a bipartite digraph. Then Alg. 1 enumerates all MR-chordless elementary circuits. Moreover, it generates each MR-chordless elementary circuits exactly once.

#### Proof

Every elementary circuit, and thus every MR-chordless elementary circuit is contained in a unique strongly connected component of *G*. It therefore suffices to consider the strongly connected component $G^{\prime }$ of *G* individually. Moreover, if $V(G^{\prime })<4$, it can contain only digons, hence it suffices to run $$\texttt {MRChordlessCircuitSearch()}$$ for all stems contained in a strongly connected components with $|V(G^{\prime })|\geq 4$. By Lemma 3, MRChordlessCircuitSearch($S,G^{\prime }$) enumerates all MR-chordless circuits containing a stem *S*. Moreover, the set of stems in $G^{\prime }$ is uniquely determined by the vertices $$r\in R\cap V(G')$$ and enumerated by Stems(). Thus Alg. 1 determines all MR-chordless circuits *C* containing *r* of length greater than 2. Lemma 1(i) ensures also that all digons containing *r* are enumerated.

To see that every MR-chordless circuits is constructed exactly once, we note that the set of digons is disjoint from the set of longer circuits produced by MRChordlessCircuitSearch(), and moreover the circuit sets for the different connected components are pairwise disjoint. Lemma 4 ensures that for each stem *S*, distinct circuits are produced. Two circuits with stems (*x*, *r*, *y*) and $\left(x^{\prime },r,y^{\prime }\right)$ with the same stem-reaction *r* are are distinct if and only if the stems are distinct.

The only remaining candidates for possible duplicate production, therefore, are two circuits *C* and $C^{\prime }$ with stems (*x*, *r*, *y*) and $\left(x^{\prime },r^{\prime },y^{\prime }\right)$ such that $r\in V(C^{\prime })$ and $r^{\prime }\in V\left(C\right)$ with $$r\ne r^{\prime}$$ Constraining the successor function such that $$r^\prime\in E(C)$$ implies $r^{\prime }>$ if *r* is the reaction-vertex in the stem used to produce *C*, thus $r^{\prime }>r$ and $r>r^{\prime }$, a contradiction. Thus the sets of cycles produced from different stems are always pairwise disjoint. □

Revisiting of reactions vertices corresponding to stems that have been exhausted previously, can be prevented by initializing the F array with 1 for these nodes. In this manner it is not necessary to fix an order for *R*. Instead, an arbitrary unused *r* is used to define the next set of stems. In our preferred implementation, however, we removed these reaction vertices from the strongly connected subgraph $G^{\prime }$ and enqueued all SCCs $G^{\prime \prime }$ of $$G'{\setminus } \{r\}$$ for subsequent processing.

### Adapting algorithm 2 for enumerating autocatalytic cores

In the following we adapt this base algorithm to enumerate MR-chordless elementary circuits for the efficient enumeration of autocatalytic cores. To this end we recall from section Theoretical background that (i) all autocatalytic cores are MR-chordless superpositions of MR-chordless elementary circuits, and (ii) no extension of an autocatalytic core can again be an autocatalytic core. As a consequence, further exploration of a path can be abandoned as soon as an MR-chordless circuit with autocatalytic sub-matrix has been identified.

We therefore modify Alg. 2 in the following way (see Alg. 3): Upon detection of an MR-chordless elementary circuit *C* in $\textbf{K}$, we retrieve the associated sub-matrix $\textbf{S}[C]$ of the stoichiometric matrix and examine its autocatalytic capacity by checking condition (A2) (see section Theoretical background), as described in [15].

**Algorithm 3:**
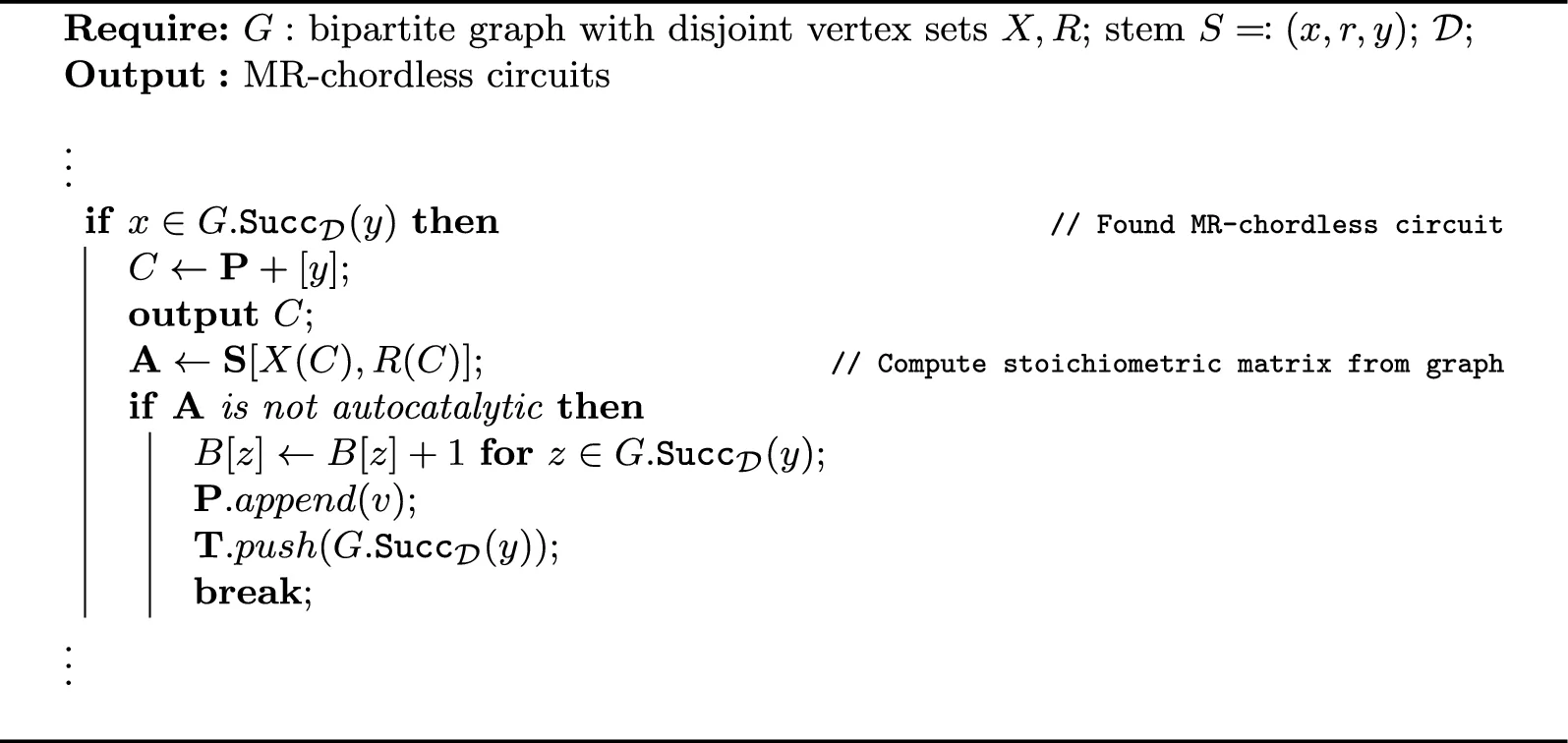
Modification of MRChordlessCircuitSearch() for autogatito.

If $\textbf{S}[C]$ is autocatalytic, $r_k$ is not appended to the current path $\textbf{P}= (x_1,r_1, \ldots , x_k)$, and the next potential neighboring reaction vertex of $x_k$ is considered. Otherwise, $r_k$ is appended to $\textbf{P}$ and the search continues as described above. This avoids the enumeration of non-core autocatalytic Metzler matrices for which a smaller autocatalytic core has already been found. These non-autocatalytic MR-chordless elementary circuits are subsequently used for the assembly of *fluffles* to obtain autocatalytic cores comprising more than one elementary circuit using Alg. 1 of [15].

## Benchmarking

The benchmarking of the presented algorithm follows a two-step approach. First, we compare the efficiency of our algorithm as a stand-alone Python implementation (MRChordlessCircuits.py) against Johnson’s well-established algorithm for enumerating all elementary circuits in two different graph models: i) random graphs of different sizes and connectedness, and ii) in fluffles, where all elementary circuits are MR-chordless to warrant equal conditions. Secondly, we include the algorithm into a Python package autogatito for enumerating autocatalytic cores. We remark at this point that autogatito builds on the Python package autogato, which has been published previously [15].

### Enumeration of MR-chordless cycles

To evaluate the efficiency of our algorithm for enumerating MR-chordless circuits in bipartite graphs, we compared it as a stand-alone Python implementation (MRChordlessCircuits.py) to Johnson’s algorithm for the enumeration of elementary circuits with a post-processing step that identifies MR-chords and terminates as soon as an MR-chord has been detected.

**Fig. 3:**
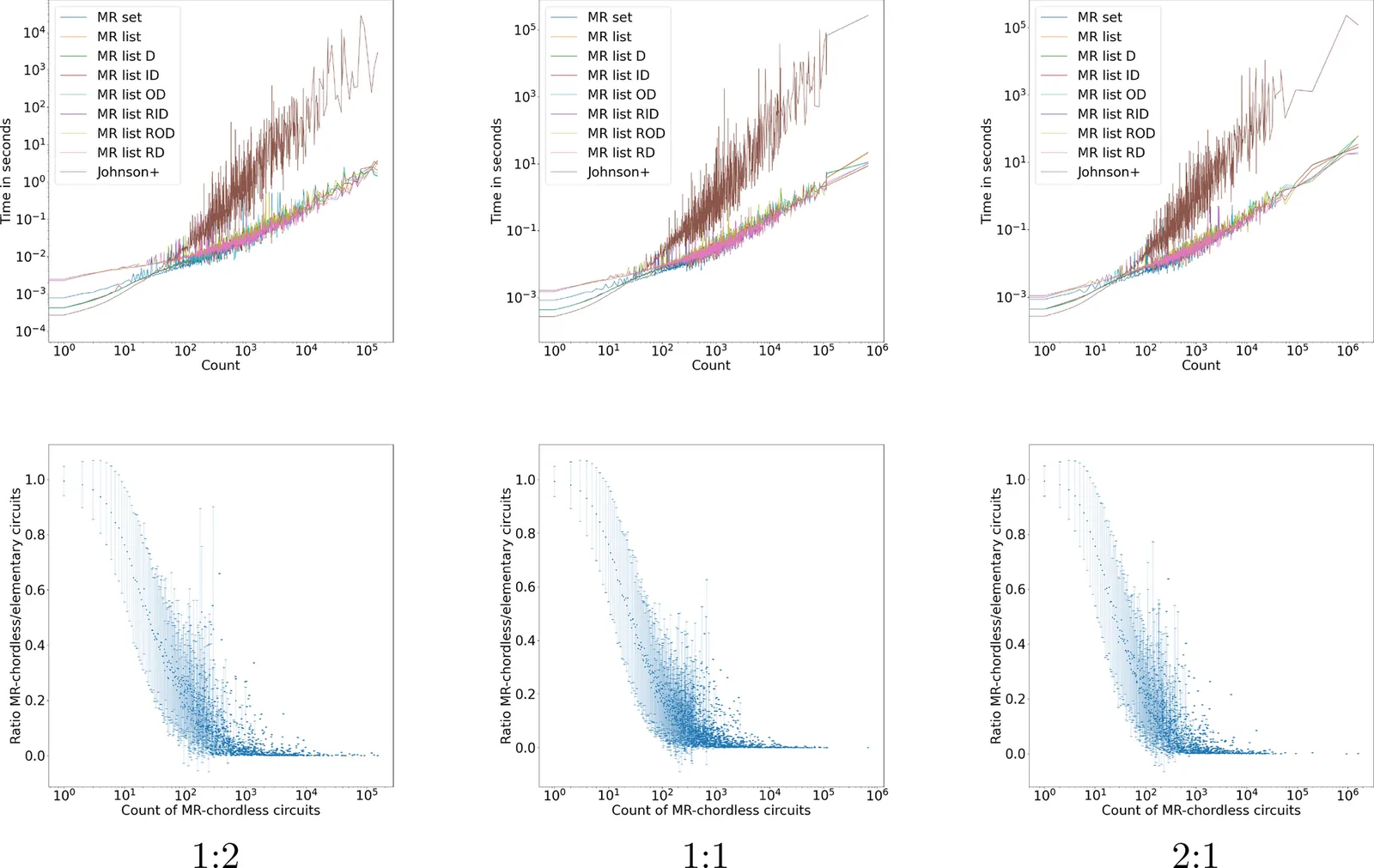
Comparison of the MR-chordless algorithm with Johnson’s algorithm to enumerate all elementary circuits, including post-processing w.r.t. required enumeration time **(top)** and the ratio of MR-chordless to all elementary circuits **(bottom)** for random bipartite graphs of a fixed ratio of metabolite and reaction vertices up to sizes of 150 and maximum edge probabilities of 2.5%. ID (RID), OD (ROD), and D (RD) specify the selective method for choosing a reaction vertex of the currently considered strongly connected component in Alg. 1. In particular, for ID, OD, and D, the reaction vertex with maximum in-, out-, and total degree, respectively, in the König graph is chosen, while for RID, ROD, and RD, the reaction vertex with the highest in-, out-, and total degree, respectively, in the R-Graph is chosen. Johnson+ refers to enumeration of all elementary circuits with Johnson’s algorithm and additional post-processing for identifying the MR-chordless among those

As a test set, we generated random bipartite Erdős-Rényi graphs of different sizes, ratios of vertex set sizes, and edge probabilities engaging NetworkX’s random-bipartite graph generator [25, 26]. Ratios of metabolites to reactions were 2 : 1, 1 : 1, and 1 : 2 while the total size of the graphs encompassed 30, 60, 90, 120, and 150 vertices with edge probabilities ranging from 0.5% to 2.5%. In the description of Alg. 2, successors of a vertex are treated as sets returned by the function $\texttt {Succ}_\mathcal {D}()$. This choice was made to achieve the optimal theoretical complexity, as sets allow *O*(1) search and deletion of elements. Yet, in practical terms hashing of every member creates a large overhead. As successor sets are generally very small, i.e., $|\texttt {Succ}_\mathcal {D}()| \ll |X|$, linear search and deletion costs may be lower than hashing costs. Hence, both lists and sets were tested in the implementation. As expected, for all three algorithms, time requirements grow exponentially. Nevertheless, our new algorithm outperformed Johnson’s enumeration of all elementary circuits with post-processing by up to 4 orders of magnitude for graphs surpassing $10^4$ MR-chordless elementary circuits. In addition, we did not observe a significant difference in the running-times of the algorithm when using a set or a list to determine the successors of a vertex.

To improve the performance of our algorithm we also investigated different orderings of reaction vertices according to their degree in the bipartite graph and in the associated R-graph. To this end, we selected for each strongly connected component *G* the vertex with the maximum in-degree, out-degree, or total degree (i.e., the sum of in- and out-degree) in the bipartite Kőnig graph or the R-graph, i.e., the digraph with vertex set *R*(*G*) and edge-set $F:=\{(r,q) \ | \ \exists x\in X(G): s^+_{xr}>0\,\wedge \, s^-_{xq}>0\}$. However, none of these modifications showed a clear performance improvement in comparison to choosing a reaction vertex at random in random bipartite Erdős-Rényi graphs (see Fig. 3, upper panels). However, an advantage might only be detectable for more densely connected graphs.

Clearly, the stronger performance of our algorithms can be primarily attributed to the growing number of elementary circuits in contrast to MR-chordless circuits (see Fig. 3, lower panels). As observed for autocatalytic cores, growing network size and increasing interconnectivity increases the likelihood for MR-chords within elementary circuits, naturally limiting the number of MR-chordless circuits and contributing to the strong divergence between the number of elementary circuits and MR-chordless elmentary circuits.

**Fig. 4:**
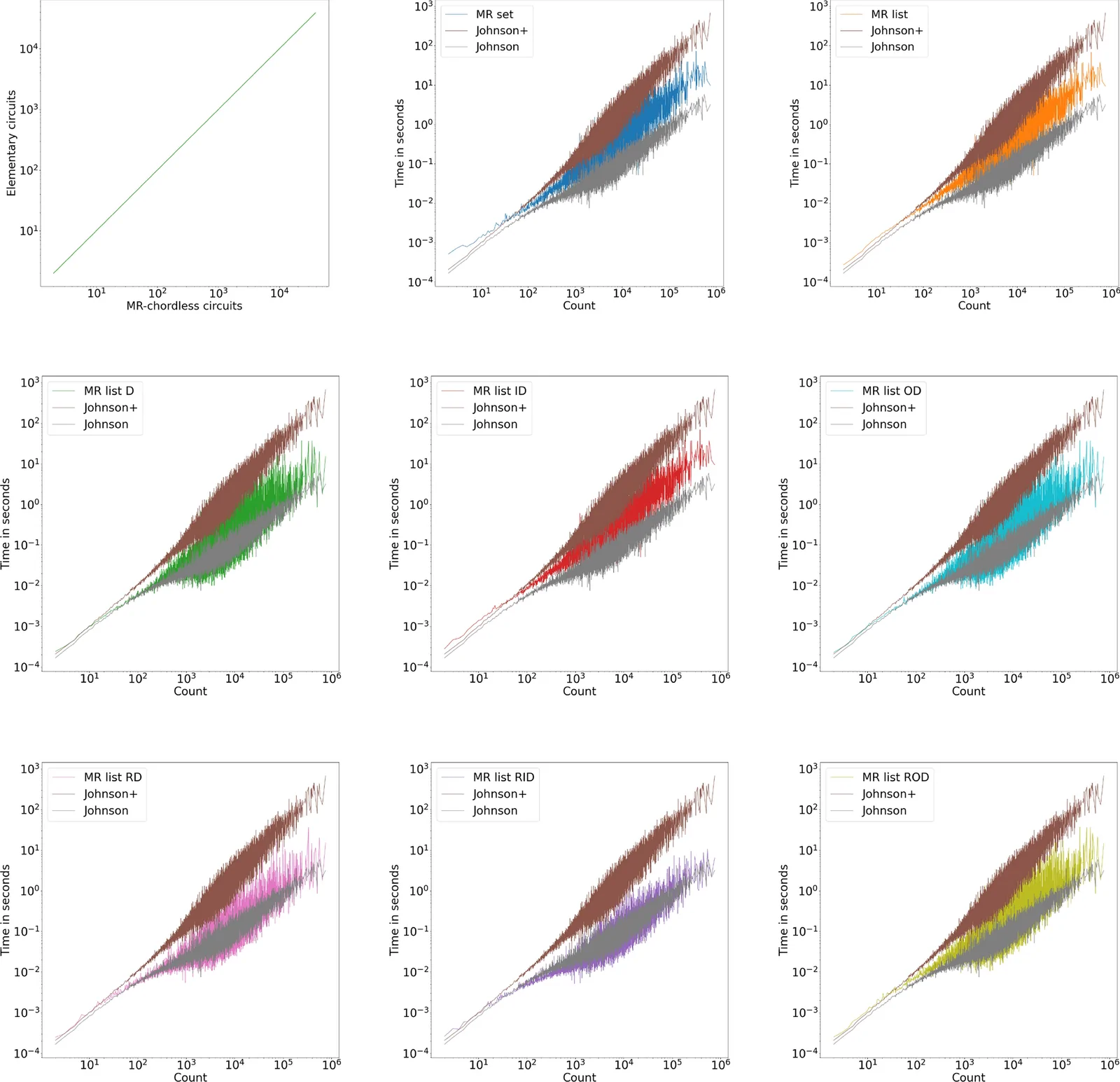
Comparison of Johnson’s algorithm and our algorithm to enumerate all elementary circuits enumerated in randomly generated bipartite fluffle graphs as described, with up to 25 attached ears of sizes ranging from 2–20. **Upper right:** Count of elementary circuits vs. MR-chordless elementary circuits count, time requirements for Johnson’s with (Johnson+) and without subsequent check for MR-chordlessness vs. our MR-chordless algorithm using random vertex selection for strongly connected components and a set **(upper middle)** or a list **(upper middle)** as vertex neighborhood data structure, and using a list as well as selecting the vertex with the maximum in-degree, out- and total-degree in the König graph **(middle row)** or in the R-graph **(lower row)**. All benchmarks are in reference to our Python implementation and datatypes represent the CPython defaults

In contrast to fluffles, the number of elementary circuits and MR-chordless elementary circuits are not necessarily the same in randomly generated bipartite graphs. To provide equitable conditions, we benchmarked the performance of our algorithm against Johnson’s algorithm in fluffles, which we generated randomly using the following construction, starting from an elementary bipartite circuit composed of 2 reactions and 2 metabolites. Then RM-ears, i.e., ears initiating in a reaction vertex and terminating in a metabolite vertex, are added. In each step, either a metabolite and a reaction vertex or an MR-edge is chosen at random. Subsequently, a bipartite path of even length k≥2 is inserted between the selected starting reaction and terminating metabolite node, or the MR-edge is split, and a metabolite and reaction vertex picked for the described path insertion. The value of *k* is chosen (with equal probabilities) as a random even integer between 2 and a tuneable maximum length parameter, here 20. This procedure ensures that only fluffles are produced and thus all elementary circuits are MR-chordless.

**Fig. 5:**
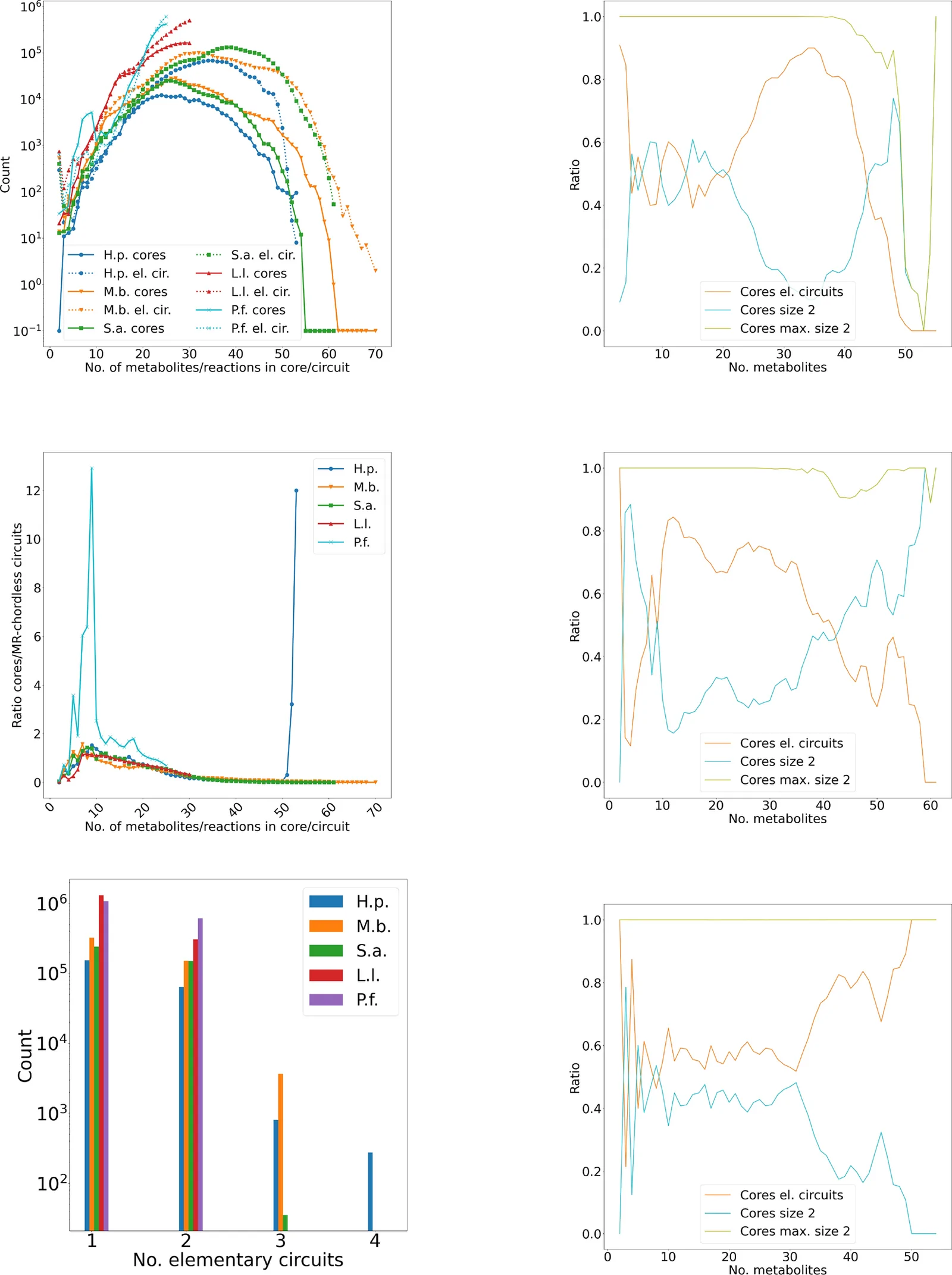
**Left panels:** Depiction of the number of MR-chordless elementary circuits and autocatalytic cores **(upper)**, the ratio of autocatalytic cores to MR-chordless elementary circuits **(middle)** depicted as a function of the indicated number of metabolite (reaction) vertices on the horizontal axis, as well as the size-distribution of the minimal circuitnets required for autocatalytic cores generation **(lower)** for metabolic networks of *Helicobacter pylori (H.p.)* [18], *Methanosarcina barkeri (M.b.)* [19], *Staphylococcus aureus (S.a.)* [20], *Lactoccus lactis (L.l)* [21], and *Plasmodium falciparum (P.f.)* [27]. **Right panels:** Ratio of autocatalytic cores that correspond to elementary circuits, the superimposition of two elementary circuits, and both of indicated size for *H.p.* **(upper)**, *M.b.* **(middle)**, and *S.a* **(lower)**

As expected and depicted in Fig. 4 (upper left panel), the number of elementary circuits computed using Johnson’s algorithm equals the number of MR-chordless elementary circuits computed using Alg. 1. Even in the case of fluffles, our algorithm outperformed Johnson’s algorithm with a subsequent check for MR-chordlessness, in particular for larger network sizes (see Fig. 4). In the case of fluffles, however, the test for MR-chordlessness is not necessary. In contrast, Johnson’s algorithm alone showed tremendously reduced time requirements for the enumeration of MR-chordless elementary circuits in fluffles and was superior to our enumeration algorithms, where no additional vertex sorting was enforced (Fig. 4 upper middle and right panel). However, these limitations could be overcome by selecting reaction vertices for stem generation with respect to their degrees. In particular, the best performance, equal to Johnson’s algorithm, was achieved when always the vertex with the highest in-degree in the R-graph was selected (Fig. 4 lower middle panel).

### Application to Metabolic Networks

To demonstrate the usefulness of the algorithms developed in section The algorithm, we compared autogatito with autogato’s core enumeration algorithm [15]. Figure 5 summarizes the results for the metabolic networks used in Fig. 1. In contrast to autogato, which depends on the enumeration of all elementary circuits, autogatito was able to enumerate all MR-chordless elementary circuits independent of their size and obtained a complete list of all autocatalytic cores that are represented by a single elementary circuit directly for *Helicobacter pylori* [18], *Methanosarcina barkeri* [19], and *Staphylococcus aureus* [20]. Moreover, for these species, we were also able to enumerate all autocatalytic cores that are superimpositions of non-autocatalytic MR-chordless elementary circuits. For all other species depicted, autogatito significantly increased the size (e.g. doubled for *Lactococcus lactis*) of all enumerated autocatalytic cores represented by MR-chordless circuits and their superimpositions. Thus, the number of elementary circuits necessary to detect the same number of autocatalytic cores of a particular size was reduced dramatically (Fig. 5, upper left panel).

The middle left panel of Fig. 5 shows the ratio of MR-chordless elementary circuits to autocatalytic cores. Surprisingly, the majority, (70% in H.p.  67% in M.s., and 61% in S.a.), of all autocatalytic cores were represented by elementary circuits. The remainder was composed mainly of two elementary circuit. In particular, more than 99% of the autocatalytic cores are either an elementary circuit or a superimposition of two elementary circuits (Fig. 5, right panels). We note, however, that even if an autocatalytic core was induced by a single elementary circuit, the induced fluffle might exhibit more elementary circuits (see e.g. a type V core with 5 elementary circuits). We, thereby, always refer to the size of the smallest circuit-net, i.e., the minimum number of elementary circuits, required for composing the particular autocatalytic core.

**Fig. 6:**
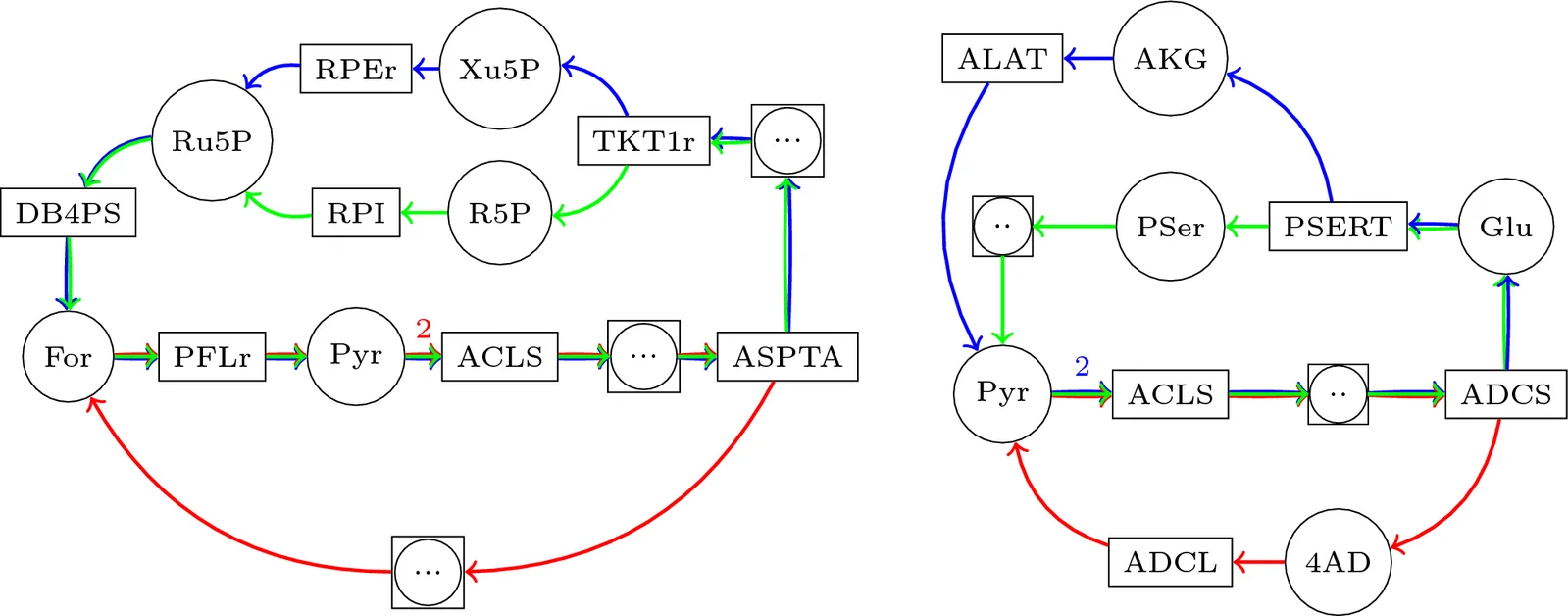
Simplified depiction of two autocatalytic core of in total 23 **(left)** and 35 **(right)** metabolites and reactions from the metabolic network of *Staphylococcus Aureus* [20] composed of 3 elementary circuits, depicted in red, green, and blue. Metabolites are depicted with circles and reactions as squares, and an alternating sequence of metabolites and reactions as one symbol composed of a square and a circle. If not otherwise specified above the edges, stoichiometries are trivial. Depicted metabolites comprise For: Formiate, Pyr: pyruvate, Xu5P: xylulose 5-phosphate, R5P: ribose 5-phosphate, Ru5P: ribulose 5-phosphate, Glu: glutamate, Pser: phosphoserine, AKG: α-ketoglutarate, 4AD: 4-amino-4-deoxychorismate and reactions PFLr: Pyruvate formate lyase reverse, ACLS: Acetolactate-lyase, ASPTA: Aspartate transaminase, TKT1r: Transketolase-1 reverse, RPEr: Ribulose 5-phosphate 3-epimerase reverse, RPI: Ribose-5-phosphate isomerase, DB4PS: 3,4-Dihydroxy-2-butanone-4-phosphate synthase, ADCS: 4-amino-4-deoxychorismate synthase, ADCL: 4-aminobenzoate synthase, PSERT: Phosphoserine transaminase

A classification of autocatalytic cores in *fully reversible* networks stipulates the existence of exactly five distinct classes [10], consisting of either a single elementary circuit or a superpositions of two circuits (type III). Here, we consider the general case of RNs composed of both reversible and irreversible reactions. The structure of autocatalytic cores in this setting may be more complex. Indeed, our computational survey uncovers a small number of such more complex autocatalytic cores that appear as superpositions of three, and in a few cases even four elementary circuits, as shown in the lower-left panel in Fig. 5.

Figure 6 shows two autocatalytic cores closely resembling a type III core. Since acetolactate-synthetase requires two pyruvate molecules and all other stoichiometries are trivial, two elementary circuits are not sufficient for the corresponding fluffle to be autocatalytic. In both cases, the upper wing is composed of two elementary circuits. The additional elementary circuit (green) shares the common MR-path of the other two as well as additional paths with the blue circuit, and thereby compensates for the non-trivial educt stoichiometry of pyruvate in acetolactate synthesis.

**Fig. 7:**
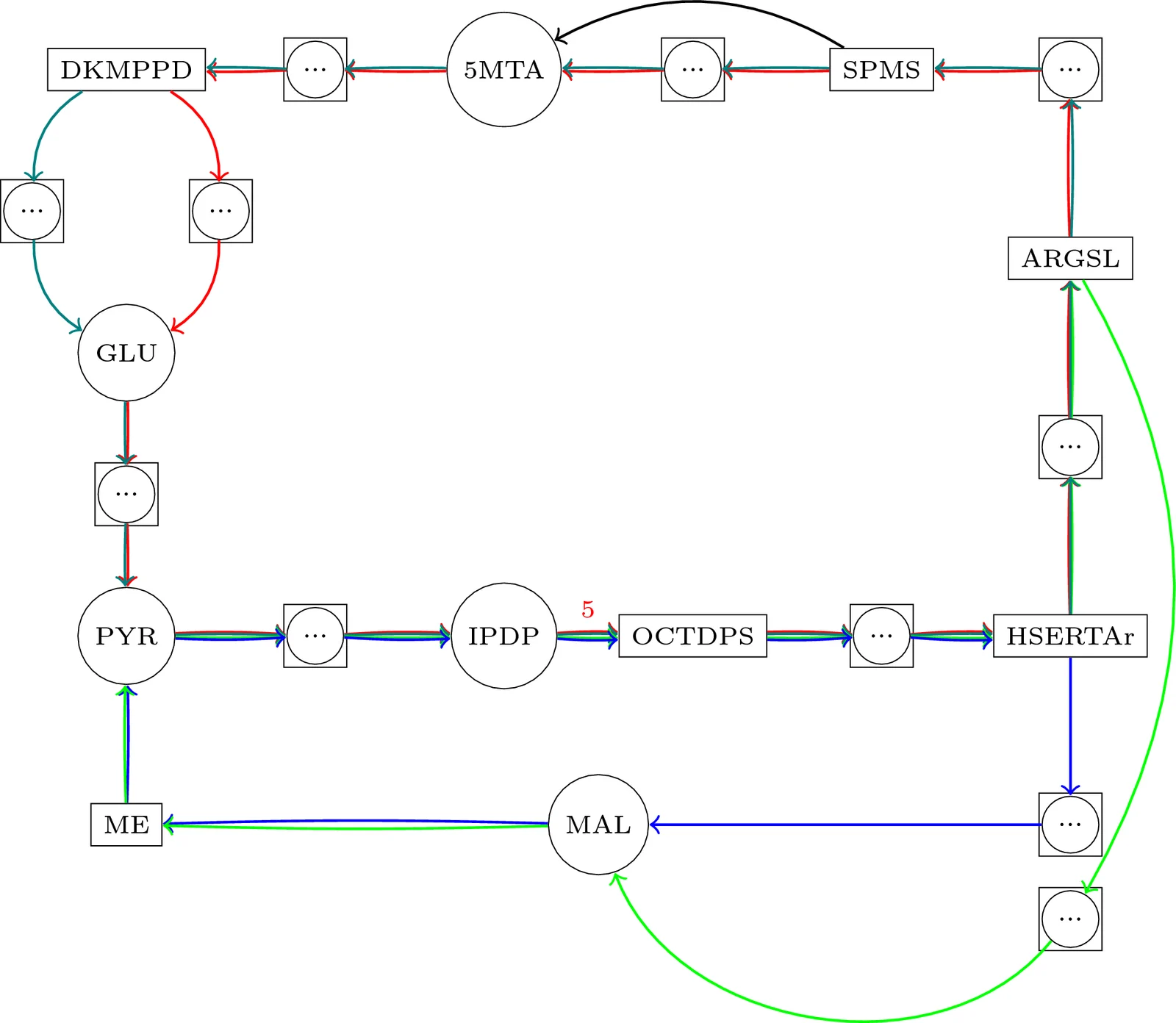
Simplified depiction of an autocatalytic core composed of 40 metabolites and 40 reactions from the metabolic network of *Helicobacter pylori (H.p.)* [18] composed of 4 elementary circuits, blue, red, green, and teal. Metabolites are depicted as circles and comprise PYR: pyruvate, GLU: glutamate, IPDP: Isopentenyl-diphosphate, MAL: Malate, 5MTA: 5-methylthioadenosine, while reactions are depicted as rectangles and comprise ME: malate-enzyme, ARGSL: argininosuccinate lyase, DKMPPD: 2,3-diketo-5-methylthio-1-phosphopentane degradation reaction, HSERTAr: homoserine O trans acetylase reverse, OCTDPS: octaprenyl pyrophosphate synthase, SPMS: spermidine synthase. An alternating sequence of metabolites and reactions is depicted as a square and a circle in one symbol

Figure 7 shows an even more complex example of an autocatalytic core formed by four elementary circuits. Again, the composed fluffle exhibits many similarities with a type III core from [10]. All elementary circuits share a common central MR-path. However, in addition to an elementary circuit connecting the upper and lower part (green), the upper wing is composed of two elementary circuits which are both doubled in their productivity by an additional RM-shortcut, which is essential to compensate for the 5 isopentenyl-diphosphate molecules necessary for octaprenyl-pyrophosphate synthesis, in order to ensure that the emerging fluffle is autocatalytic.

## Concluding remarks

Autocatalytic cores are an important characteristic of chemical reaction networks and may serve as a key discriminating feature between the metabolic networks of living organism and inanimate chemical systems. The algorithmic advances described here make it possible to obtain the (nearly) complete inventory of autocatalytic cores for large graphs, thus enables a systematic investigation of autocatalysis in chemical reaction network data. In our own benchmarks we enumerated datasets comprising up to $3\cdot 10^7$ MR-chordless circuits and $10^7$ autocatalytic cores. Owing to the high memory overhead of Python data-structures and other language restrictions, the current implementation is limited mainly by its RAM requirements. This, unfortunately, also limits parallelization, as RAM usage increases linearly with the number of subprocesses. Hence, computations were distributed to set of servers with each 512GB of RAM.

Empirically, we observe that the overwhelming majority of autocatalytic cores are either formed by a single MR-chordless elementary circuit or constitute the superposition of a pair of such circuits. Structurally, these cases conform the classification of autocatalytic cores in *fully reversible* networks [10]. For very large networks, the limiting resource is the memory required to store the elementary MR-chordless circuits that are not autocatalytic for the second stage of autogatito, i.e., for testing autocatalysis of pairwise or even larger combinations. We observe, however, that the majority of autocatalytic cores are formed by a single elementary circuit in the König graph $\textbf{K}$. Enumeration of MR-chordless elementary circuits thus provides a reasonable approach towards characterizing autocatalysis, even for network sizes that render the exhaustive generation of larger autocatalytic cores via superimposition of MR-chordless elementary circuits computational infeasible.

An important result of our computational survey in real-life metabolic networks is the fact that autocatalytic cores beyond the classification given in [10] do exist if irreversible reactions are included. Although formally all chemical reactions are reversible, the differences in free energies between reactants and products is so large in many cases that the ratio of product to reactant concentrations required to drive the reaction energetically “up-hill” are not achievable in practice. The capability to handle irreversible reactions, therefore, is of key practical relevance. This begs the question of whether autocatalytic cores can at least in principle become arbitrarily complex in partially irreversible RNs, or whether there is a finite list of discernible types.

Complete metabolic networks are likely to contain much larger sets of reactions than the models used here. The Human Metabolome Database [28], for example, lists more than 200,000 substances as of Jan. 2026. Since the number of (long) elementary circuits increases exponentially with size of the König graph $\textbf{K}$, complete enumerations are bound to fail eventually as larger and larger network models become available. Nevertheless, it will remain feasible to list MR-chordless elementary circuits at least up to a certain maximum length, storing the non-autocatalytic ones on disc. Such a strategy will then require dedicated algorithms to efficiently identify composite autocatalytic cores composed of two or more elementary circuits. While autogatito already performs well on contemporary datasets, we plan a low-level implementation to improve on memory consumption and parallelization in future.

## Supplementary Information

Supplementary material 1.

## Data Availability

The implementation, models and all necessary data is available at https://github.com/hollyritch/MRChordlessCircuits.
