# Supplementary material for: Using MR-chordless circuits for efficient enumeration of autocatalytic cores in large chemical reaction networks
Source: J Cheminform. 2026 Aug 20;18:117. doi: 10.1186/s13321-026-01240-3 (PMC13491814; doi:10.1186/s13321-026-01240-3)
Supplement: Supplementary file 1 [file 13321_2026_1240_MOESM1_ESM.pdf]

Using MR-Chordless Circuits for Efficient Enumeration of Autocatalytic Cores in Large Chemical Reaction Networks
Supplementary Information

Richard Golnik[1,3*], Thomas Gatter[1], Nicola Vassena[1], Peter F. Stadler[1,2,3,4,5,6,7]

[1]Bioinformatics Group, Department of Computer Science Leipzig University, Härtelstraße 16–18, D-04107 Leipzig, Germany.
[2]Interdisciplinary Center for Bioinformatics & Zuse School for Embedded and Composite Artificial Intelligence (SECAI) & Center for Scalabale Data Analytics and Artificial Intelligence, Leipzig University, D-04107 Leipzig, Germany.
[3]Max Planck Institute for Mathematics in the Sciences, Inselstraße 22, D-04103 Leipzig, Germany.
[4]Department of Theoretical Chemistry, University of Vienna, Währingerstraße 17, A-1090 Wien, Austria.
[5]Facultad de Ciencias, Universidad National de Colombia, Bogotá, Colombia.
[6]Center for non-coding RNA in Technology and Health, University of Copenhagen, Ridebanevej 9, DK-1870 Frederiksberg, Denmark.
[7]Santa Fe Institute, 1399 Hyde Park Rd., Santa Fe, NM 87501, USA.

*Corresponding author(s). E-mail(s): richard@bioinf.uni-leipzig.de;
Contributing authors: thomas@bioinf.uni-leipzig.de; nicola.vassena@uni-leipzig.de; studla@bioinf.uni-leipzig.de;

1 Graph Theory

A graph $G = (V, E)$ is a tuple composed of a set of vertices V and a set of edges E. In case of undirected graphs, E contains unordered pairs of vertices $\{u, v\}$ whenever $u \in V$ is adjacent to $v \in V$ or vice-versa. For directed graphs, also referred to as digraphs, E consists of ordered pairs, thus $(u, v) \in E$ if and only if there is an edge from u to v. The underlying undirected graph of a digraph G is the graph $G_\circ$ with vertex set $V(G)$ and edge set $\{\{u, v\} \mid (u, v) \in E(G)\}$.

In undirected graphs, the *neighborhood* of a vertex v is defined as the set of its adjacent vertices. In pseudo-code notation:

$$G.neighbors(v) = \{u \mid \{v, u\} \in E(G)\}.$$

Accordingly, the *degree* of v is defined as the cardinality of the neighborhood: degree(v)=$|G.neighbors(v)|$. In directed graphs, predecessors

$$G.pred(v) = \{u \mid (u, v) \in E(G)\} \tag{1}$$

and successors

$$G.succ(v) = \{u \mid (v, u) \in E(G)\} \tag{2}$$

of a node v are distinguished. Accordingly, the *in- and out-degree* of v are the respective cardinalities, i.e.,

$$\text{in-degree(v)} = |G.pred(v)| \qquad \text{out-degree(v)} = |G.succ(v)|.$$

For undirected graphs, a *path* is a sequence of distinct vertices $(v_1, \ldots, v_k)$ such that $\{v_i, v_{i+1}\} \in E$. The graph is called *connected* if for any pair of vertices $u, v \in V$ there is a path from u to v or vice versa. It is called *2-connected* if for any three vertices $v, u_1, u_2 \in V$ there exists a path connecting u_1 to u_2, which does not include v. Informally, 2-connectedness means that the graph is still connected upon the removal of any of its vertices.

In turn, a directed graph G is called *connected* (resp. *2-connected*) if its underlying undirected graph $G_\circ$ is connected (resp. 2-connected). The definition of a directed path in a digraph G is analogous to the undirected case: a *directed path* is a sequence of distinct vertices $(v_1, \ldots, v_k)$ such that $(v_i, v_{i+1}) \in E$, that is, the path follows the orientation of the edges. A digraph is called *strongly connected* if for each pair of vertices $u, v \in V$ there is a directed path connecting u to v and a directed path connecting v to u. From this point of view, a connected digraph is also said to be weakly connected. If G is strongly and 2-connected it is called a *strong block* (or strongly connected block).

From now on, we solely focus on digraphs $G \coloneqq (V, E)$. A digraph $G' \coloneqq (V', E')$ with vertices $V' \subseteq V$ and edges $E' \subseteq E$ is called a *subgraph* of G, denoted as $G' \subseteq G$. If G' is strongly connected then G' is called a strongly connected subgraph, and if G' is, in addition, 2-connected then G' is called a strong block of G. G' is said to be *induced* by V' if for $u, v \in V'$ with $(u, v) \in E$ it always follows that $(u, v) \in E'$. In this case, we write $G' = G[V']$ for the subgraph induced by V'. A maximal set

of vertices $W \subseteq V$ such that the induced subgraph $G[W]$ is strongly connected is a *strongly connected component*. If $V = W$, then G itself is strongly connected, i.e., it possesses only one trivial connected component, the whole graph itself.

An *elementary circuit* C in a directed graph G is a sequence of vertices $(v_1, \ldots, v_k, v_1)$ such that $(v_1, \ldots, v_k)$ is a path and $(v_k, v_1) \in E$. We denote by $V(C)$ and $E(C)$ the vertices and edges of C. A *chord* of an elementary circuit C is an edge $(v_j, v_k) \in E$ such that $v_j, v_k \in V(C)$ but $k \neq j+1$, i.e., $(v_j, v_k) \notin E(C)$. Note that - by definition - any two vertices u and v in a strong block of a directed graph G belong to at least one common elementary circuit C.

A *directed bipartite graph* $G = (V, E)$ is a special instance of digraph, where the set V admits a partition into two disjoint sets of vertices $V = X \cup R$ and $X \cap R = \emptyset$, such that there are no edges connecting vertices belonging to the same set. In particular, then, the partition of the vertex set $V = X \cup R$ induces a natural partition of the edge set $E = E_1 \cup E_2$, where $(u, v) \in E_1$ if $u \in X$ and $v \in R$ and $(u, v) \in E_1$ if $u \in R$ and $v \in X$. As a directed bipartite graph is, in particular, a digraph, all of the above notions apply identically to the bipartite case.

2 Metabolic Networks

Metabolic networks in SBML format were downloaded as .xml-files from the BIGG database [1] and from each network a set of small molecules with high connectivity removed, co-factors, catalysts, as well as energy providing or electron transferring molecules. The overall list of molecules fed to *autogatito* was derived from the union of species from all metabolic networks of the BIGG-database. However, since not each metabolite is present in each metabolic network, the list of removed molecules may differ from species to species. In each case, we provide here the list of BIGG-identifiers and refer to the BIGG-website for complete names of the chemical molecules. The prefix "M" always refers to metabolite and the suffixes "c", "e", "g" "h", "m", and "r" to the compartments, cytosol, extracellular, golgi apparatus, chloroplast, mitochondrial, and endoplasmic reticulum, respectively.

2.1 *Helicobacter pylori*

The set of species removed from the metabolic network of Helicobacter pylori [2] comprised M_fmn_c, M_dctp_c, M_ppi_c, M_pro__L_e, M_fadh2_c, M_na1_e, M_pi_c, M_h2s_c, M_pa_Hp_c, M_fum_e, M_succ_e, M_ump_c, M_gmp_c, M_dtmp_c, M_ctp_c, M_dttp_c, M_aa_e, M_imp_c, M_ribflv_c, M_so3_c, M_cdp_c, M_datp_c, M_core_lps_hp_c, M_dcdp_c, M_dump_c, M_mqn6_c, M_hco3_c, M_dgmp_c, M_dutp_c, M_trnaglu_c, M_h2o_c, M_damp_c, M_h2co3_e, M_pime_e, M_dudp_c, M_mql6_c, M_gdp_c, M_h2_e, M_o2_c, M_h2co3_c, M_cmp_c, M_ala__L_e, M_co2_c, M_na1_c, M_dadp_c, M_atp_c, M_nadph_c, M_no_c, M_thmpp_c, M_ACP_c, M_nh4_c, M_so4_c, M_nad_c, M_adp_c, M_dnad_c, M_dgdp_c, M_dcamp_c, M_no3_c, M_fmnh2_c, M_nmn_c, M_no2_c, M_fe2_c, M_nadp_c, M_nadh_c, M_nmn_e, M_h_c, M_h2o2_c, M_coa_c, M_btn_c, M_o2s_c, M_udp_c, M_fad_c, M_utp_c, M_amp_c, M_dgtp_c, M_gtp_c, M_thmmp_c, and M_h_e.

2.2 *Methanosarcina barkeri*

The set of species removed from the metabolic network of Methanosarinca barkeri [3] comprised M_trnaleu_c, M_ump_c, M_f430p1_c, M_h2o_c, M_dctp_c, M_k_c, M_cobya_c, M_ch4_e, M_imp_c, M_actn__R_e, M_k_e, M_trnalys_c, M_o2s_c, M_so3_e, M_cl_c, M_fmn_c, M_com_c, M_thm_c, M_cmp_c, M_f390a_c, M_udp_c, M_h_c, M_co2dam_c, M_meoh_e, M_mg2_c, M_f420_0_c, M_trnahis_c, M_ppi_c, M_f390g_c, M_idp_c, M_damp_c, M_trnaser_c, M_dcdp_c, M_co_c, M_trnaala_c, M_cob_c, M_nmn_c, M_nadph_c, M_dcmp_c, M_trnaglu_c, M_datp_c, M_ribflv_c, M_cbi_e, M_cdp_c, M_pppi_c, M_tma_e, M_gmp_c, M_ala__L_e, M_cobalt2_c, M_itp_c, M_ni2_c, M_adp_c, M_dnad_c, M_gtp_c, M_cd2_e, M_f420_1_c, M_dma_e, M_mma_e, M_dtmp_c, M_trnaile_c, M_trnagln_c, M_pac_e, M_na1_e, M_co2_c, M_btn_e, M_cu2_c, M_dump_c, M_trnatrp_c, M_ca2_e, M_thmpp_c, M_fe2_c, M_cu2_e, M_f420_2_c, M_pi_c, M_unknown_cbl1deg_e, M_coa_c, M_h_e, M_atp_c, M_trnamet_c, M_dgdp_c, M_f420_5_c, M_ctp_c, M_f420_6_c, M_no2_c, M_cbl1hbi_e, M_n2_e, M_nadp_c, M_h2o2_c, M_dgtp_c, M_alac__S_e, M_dudp_c, M_ca2_c, M_trnaasp_c, M_dcamp_c, M_dutp_c, M_dadp_c, M_f420_7_c, M_h2_e, M_thmmp_c, M_cl_e, M_f420_2h2_c, M_trnaphe_c, M_na1_c, M_trnapro_c, M_trnatyr_c, M_cd2_c, M_f430p2_c, M_dms_e, M_btn_c, M_trnagly_c, M_trnacys_c, M_utp_c, M_h2_c, M_s_e, M_ch4s_e, M_dtdp_c, M_ind3ac_e, M_s_c, M_trnaarg_c, M_co1dam_c, M_f420_4_c, M_nh4_c, M_f420_3_c, M_camp_c, M_trnathr_c, M_hco3_c, M_nad_c, M_gdp_c, M_trnaval_c, M_o2_c, M_unknown_rbfdeg_e, M_h2s_c, M_glyald_e, M_f430p3_c, M_so3_c, M_nadh_c, M_f430_c, M_tsul_c, M_amp_c, and M_dttp_c.

2.3 *Staphylococcus aureus*

The set of species removed from the metabolic network of Staphylococcus aureus [4] comprised M_h_c, M_trnagly_c, M_thmmp_c, M_cdp_c, M_fmn_c, M_dgmp_c, M_dctp_c, M_mqn8_c, M_amp_c, M_nadh_c, M_nadp_c, M_dgtp_c, M_no3_c, M_datp_c, M_itp_c, M_pi_e, M_fadh2_c, M_h2s_c, M_btn_c, M_nad_c, M_dtmp_c, M_pppi_c, M_glyc3p_e, M_apoACP_c, M_atp_c, M_mql8_c, M_gmp_c, M_pro__L_e, M_udp_c, M_dump_c, M_fad_c, M_nadph_c, M_fe2_c, M_dudp_c, M_dadp_c, M_hco3_c, M_ncam_e, M_dcamp_c, M_na1_c, M_gdp_c, M_trnaser_c, M_fmnh2_c, M_o2_c, M_dcmp_c, M_arg__L_e, M_ump_c, M_na1_e, M_dcdp_c, M_trnaala_c, M_h2o2_c, M_ribflv_c, M_dttp_c, M_idp_c, M_trnaile_c, M_s_c, M_so3_c, M_pi_c, M_dnad_c, M_damp_c, M_dgdp_c, M_ACP_c, M_trnalys_c, M_adp_c, M_trnaglu_c, M_dutp_c, M_imp_c, M_utp_c, M_trnamet_c, M_ctp_c, M_ppi_c, M_cmp_c, M_trnaasp_c, M_thmpp_c, M_ptrc_e, M_trnaasn_c, M_acnam_e, M_h_e, M_no2_c, M_orn_e, M_trnaarg_c, M_btn_e, M_gtp_c, M_dtdp_c, M_cu2_c, M_nh4_c, M_coa_c, M_q8h2_c, M_o2s_c, M_rbflvrd_c, M_so4_c, M_cu2_e, M_glyald_e, M_nh4oh_c, M_q8_c, M_co2_c, M_h2o_c, M_trnathr_c, and M_trnacys_c.

2.4 *Lactococcus lactis*

The set of species removed from the metabolic network of Lactococcus Lactis [5] comprised M_pi_c, M_cellb_e, M_o2s_c, M_trnaval_c, M_trnapro_c, M_acp_c, M_amp_c, M_dttp_c, M_trnaasn_c, M_arg__L_e, M_trnaarg_c, M_glc__D_e, M_trnaleu_c,

M_thmpp_c, M_h2o2_c, M_mqn8_c, M_glu__L_e, M_hco3_c, M_trnaala_c, M_trnaasp_c, M_utp_c, M_ACP_2_c, M_PROT_LLA_c, M_actn__R_e, M_ppi_c, M_apoACP_c, M_PROT_LLA_v3_c, M_dgmp_c, M_dtmp_c, M_4abut_e, M_trnahis_c, M_dcdp_c, M_nmn_c, M_itp_c, M_CPS_LLA_c, M_gtp_c, M_dump_c, M_glyc3p_e, M_trnagln_c, M_dtdp_c, M_nadph_c, M_pa_LLA_c, M_trnathr_c, M_mql7_c, M_ribflvRD_c, M_idp_c, M_dgtp_c, M_o2_c, M_trnagly_c, M_dutp_c, M_ump_c, M_nadp_c, M_dgdp_c, M_nh4_c, M_trnaglu_c, M_ribflv_c, M_co2_c, M_atp_c, M_dadp_c, M_hqn_c, M_acgala_e, M_dudp_c, M_cdp_c, M_ctp_c, M_nmn_e, M_h2s_c, M_cmp_c, M_trnaile_c, M_thm_c, M_trnacys_c, M_dnad_c, M_pppi_c, M_adp_c, M_gdp_c, M_dcamp_c, M_h_e, M_ACP_c, M_ch4s_e, M_fadh2_c, M_trnatyr_c, M_fad_c, M_trnaser_c, M_nadh_c, M_damp_c, M_dctp_c, M_h2o_e, M_trnalys_c, M_dimp_c, M_udp_c, M_nh3_c, M_orn__L_e, M_fmn_c, M_gmp_c, M_h2o_c, M_trnamet_c, M_datp_c, M_mqn7_c, M_thmmp_c, M_diact_e, M_nad_c, M_pi_e, M_dcmp_c, M_coa_c, M_imp_c, M_trnatrp_c, M_trnaphe_c, M_RNA_LLA_c, and M_h_c.

2.5 *Plasmodium falciparum*

The set of species removed from the metabolic network of Plasmodium falciparum [6] comprised M_h_e, M_mqn4_c, M_o2s_l, M_amp_m, M_ca2_c, M_imp_c, M_ca2_e, M_datp_c, M_hco3_c, M_camp_c, M_hco3_h, M_35cimp_c, M_ribflv_c, M_q8h2_m, M_cdp_c, M_dttp_c, M_Lcyst_e, M_lys__L_e, M_h_g, M_pa_pf_18_0_20_4_c, M_dttp_e, M_nad_c, M_atp_m, M_dudp_c, M_o2s_m, M_4hba_e, M_pa_pf_16_0_16_0_c, M_fmn_c, M_dcmp_c, M_dsbcrd_r, M_gmp_c, M_mql4_m, M_ppi_h, M_udp_c, M_coa_h, M_dimp_c, M_no3_c, M_mqn4_m, M_nadh_h, M_Hb_l, M_h_m, M_asn__L_e, M_h2o_l, M_amp_c, M_ser__L_e, M_ppi_c, M_pa_pf_18_0_18_1_c, M_nad_h, M_nh4_c, M_dsbgox_r, M_nadh_m, M_no_c, M_h2o_h, M_dnad_c, M_no3_m, M_thm_e, M_nadp_h, M_apoACP_h, M_o2_m, M_dadp_c, M_atp_c, M_gdp_c, M_asp__L_e, M_dtmp_c, M_35cgmp_c, M_seln_c, M_thmpp_c, M_nadp_m, M_pa_pf_16_0_18_2_c, M_xolest_16_0_e, M_ncam_e, M_nadph_h, M_gtp_m, M_35ccmp_c, M_pi_c, M_h_l, M_dtdp_c, M_cys__L_e, M_na1_e, M_thm_c, M_chsterol_e, M_dutp_c, M_pa_pf_18_2_18_2_c, M_h2o2_c, M_dna5mtc_c, M_cmp_c, M_adp_h, M_dgtp_c, M_damp_c, M_o2s_c, M_4fe4s_h, M_btn_c, M_ctp_c, M_adp_c, M_dcamp_c, M_dcdp_c, M_pa_pf_18_1_18_2_c, M_nadp_c, M_didp_c, M_fe2_m, M_nh4_h, M_ctp_h, M_o2_l, M_nmn_c, M_cu2_e, M_arg__L_e, M_atp_h, M_fadh2_m, M_chol_e, M_ppi_m, M_leu__L_e, M_gtp_c, M_met__L_e, M_pa_pf_20_4_20_4_c, M_utp_c, M_2fe2s_h, M_na1_c, M_fad_m, M_no2_m, M_thmmp_c, M_nadph_c, M_co2_m, M_cmp_h, M_ACP_h, M_gln__L_e, M_dsbcox_r, M_co2_c, M_pppi_c, M_coa_c, M_nad_m, M_cu2_c, M_adp_m, M_ump_c, M_pa_pf_18_0_18_0_c, M_h2o2_l, M_pa_pf_16_0_20_4_c, M_glu__L_e, M_pa_pf_16_0_18_1_c, M_dna_c, M_h2o2_m, M_q8_m, M_h_c, M_dctp_c, M_nadh_c, M_h2o_g, M_pa_pf_16_0_18_0_c, M_pa_pf_18_1_20_4_c, M_gly_e, M_dgdp_c, M_ile__L_e, M_pi_h, M_gthrd_e, M_dgmp_c, M_dsbgrd_r, M_gdp_m, M_nh4_m, M_h_r, M_h2o_m, M_5fthf_e, M_fe2_c, M_gthox_e, M_h2o2_h, M_4pyrdx_e, M_ala__L_e, M_o2_c, M_co2_h, M_h_h, M_nadph_m, M_35cdamp_c, M_thr__L_e, M_dump_c, M_pa_pf_18_1_18_1_c, M_pi_m, M_no2_c, M_h2o_c, M_coa_m, M_fad_c, and M_fe2_h.

References

[1] King ZA, Lu J, Dräger A, Miller P, Federowicz S, Lerman JA, Ebrahim A, Palsson BO, Lewis NE (2016) BiGG Models: A platform for integrating, standardizing and sharing genome-scale models. Nucleic Acids Research 44(D1):D515–D522, DOI 10.1093/nar/gkv1049

[2] Thiele I, Vo TD, Price ND, Palsson BØ (2005) Expanded metabolic reconstruction of Helicobacter pylori (iIT341 GSM/GPR): An in silico genome-scale characterization of single- and double-deletion mutants. Journal of Bacteriology 187(16):5818–5830, DOI 10.1128/JB.187.16.5818-5830.2005

[3] Feist AM, Scholten JCM, Palsson BØ, Brockman FJ, Ideker T (2006) Modeling methanogenesis with a genome-scale metabolic reconstruction of Methanosarcina barkeri. Molecular Systems Biology 2:2006.0004, DOI 10.1038/msb4100046

[4] Becker SA, Palsson BØ (2005) Genome-scale reconstruction of the metabolic network in Staphylococcus aureus N315: An initial draft to the two-dimensional annotation. BMC microbiology 5:8, DOI 10.1186/1471-2180-5-8

[5] Flahaut NAL, Wiersma A, van de Bunt B, Martens DE, Schaap PJ, Sijtsma L, Dos Santos VAM, de Vos WM (2013) Genome-scale metabolic model for Lactococcus lactis MG1363 and its application to the analysis of flavor formation. Applied Microbiology and Biotechnology 97(19):8729–8739, DOI 10.1007/s00253-013-5140-2

[6] Abdel-Haleem AM, Hefzi H, Mineta K, Gao X, Gojobori T, Palsson BO, Lewis NE, Jamshidi N (2018) Functional interrogation of Plasmodium genus metabolism identifies species- and stage-specific differences in nutrient essentiality and drug targeting. PLoS computational biology 14(1):e1005,895, DOI 10.1371/journal.pcbi.1005895
